# Ultraprecision, high-capacity, and wide-gamut structural colors enabled by a mixture probability sampling network

**DOI:** 10.1038/s41377-025-02122-3

**Published:** 2026-03-11

**Authors:** Zeyong Wei, Weijie Xu, Siyu Dong, Xiaojia Liang, Jingyuan Zhu, Hui Zhang, Kaixuan Li, Lei Jin, Zhanshan Wang, Yuzhi Shi, Gang Yan, Cheng-Wei Qiu, Xinbin Cheng

**Affiliations:** 1 School of Physics Science and Engineering, Tongji University, Shanghai, China; 2 Institute of Precision Optical Engineering, School of Physics Science and Engineering, Tongji University, Shanghai, China; 3 MOE Key Laboratory of Advanced Micro-Structured Materials, Shanghai, China; 4 Shanghai Institute of Intelligent Science and Technology, Tongji University, Shanghai, China; 5 Shanghai Frontiers Science Center of Digital Optics, Shanghai, China; 6 Department of Electrical and Computer Engineering, National University of Singapore, Singapore, Singapore; 7 School of Electronics and Information Engineering, Hangzhou Dianzi University, Hangzhou, China; 8 Shanghai Eye Diseases Prevention & Treatment Center, Shanghai Eye Hospital, Shanghai, China

**Keywords:** Metamaterials, Nanophotonics and plasmonics, Displays

## Abstract

The advancement of nanophotonic devices is significantly dependent on achieving high-precision inverse design capabilities, which are critical for identifying optimal structural configurations that enable enhanced and multifunctional performances. The process of inverse design confronts a one-to-many relationship due to the complex mapping between optical performance and structure. Though several approaches, including tandem networks, mixture density networks (MDN), and conditional generative adversarial networks, have shown promising outcomes, they still face accuracy limitations when confronted with structures with higher degrees of freedom. Here, we propose a sampling-enhanced MDN called a mixture probability sampling network (MPSN), that outputs mixture Gaussian distributions (MGDs) of structural parameters through an end-to-end framework. The results of multiple samples drawn from the MGDs are fed into a pre-trained network, and the sample that minimizes the error relative to the real data is selected for network training. We benchmark the high performance in nanophotonics through the structural color design, achieving a high precision of up to 99.9% and a mean absolute error of less than 0.002. This work paves the way for resolving intricate inverse design problems in nanophotonics.

## Abstract (web summary)

We present an end-to-end mixture probability sampling network that generates Gaussian mixtures for structural parameters, achieving 99.9% precision in structural color design.

## Graphical abstract

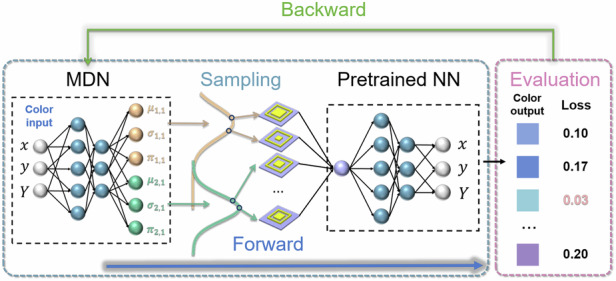

## Introduction

The rapid advancement of deep learning has ushered in a new era of breakthroughs in the inverse design of nanophotonics, particularly in the field of metasurface^1–9^. It demonstrates significant advantages in optimizing optical efficiency and device design. This probabilistic architecture enables predictive design of nanophotonic structures by inversely mapping desired optical properties to corresponding geometric parameters through deep learning. This approach significantly reduces the reliance on complex physical mechanism analysis and intensive electromagnetic simulations. Compared to traditional optimization algorithms like the adjoint method^10^, which are suitable for a specified set of targets, deep learning methods are better suited for large-scale design tasks. One of the most critical components of deep learning is the artificial neural network, which comprises a vast number of interconnected neurons that transmit information between each layer, creating nonlinear relationships between inputs and outputs within the network^11–15^. Deep learning has a broad range of applications in computer vision, natural language processing, and other fields^14^. By using the material structure as the input and the electromagnetic property as the output (forward network), the need for time-consuming electromagnetic simulations is mitigated, significantly enhancing the efficiency of optimization algorithms^3^, such as particle swarm optimization^16^. Conversely, employing the optical performance as the input and the structural parameters as the output (inverse network) can address the challenge of re-optimization when the optimization target is altered^17^. However, due to the non-uniqueness of the relationship between optical properties and structures, especially in systems with high degrees of freedom (involving multiple parameters), direct neural networks (DNNs) often face significant challenges in achieving convergence^18–21^. This inherent non-uniqueness issue critically undermines the precision of optical design frameworks, as neural network-predicted structural parameters frequently yield erroneous solutions that deviate from physical realizability.

In the past decade, numerous approaches have been proposed to address the non-uniqueness issue in metasurface design. The integrated approach combining supervised learning (SL) and reinforcement learning (DL) enables optimized structural color design across multiple parameter dimensions, merging data-driven pattern recognition with adaptive optimization strategies to navigate complex design spaces effectively^18^. This approach involves using multiple machine learning models to simplify the design process with many parameters, although obtaining sufficiently accurate solutions remains challenging. As the number of controllable parameters increases to incorporate more solutions, it becomes more difficult for the algorithm to attain an optimal outcome. To mitigate non-uniqueness issues, tandem networks (TNs) have been proposed^20^. TN connects a pretrained forward network after the inverse network, resulting in superior design results^22^. It ingeniously transforms the one-to-many problem into a one-to-one problem to ensure network convergence. The Mixture density networks (MDNs) utilize a mixture Gaussian distribution to output multiple structures^19,23^, solving the problem that TNs can only output a single solution^6,24^. There are also some other ideas called unsupervised learning models, such as generation adversarial networks^25^, conditional generative adversarial networks^21^ and variational autoencoders^26^, which can generate more solutions^24^. However, due to the use of random noise to address the one-to-many problem, the inherent uncertainty introduces some impact on the accuracy. Existing approaches other than MDN rely on specialized network architectures to circumvent the non-uniqueness problem, yet they fail to fundamentally address inabilities of networks to generate multiple solutions simultaneously. However, the performance of MDN remains constrained by its predefined distribution parameters, leading to suboptimal training outcomes.

In this work, we develop a network architecture that inherently incorporates non-uniqueness characteristics, capable of generating multiple structural-parameter sets for a single design objective while maintaining the training stability to be unaffected by the solution degeneracy. To do so, an end-to-end mixture probability sampling network (MPSN) is proposed, which generates Gaussian mixture distributions of structural parameters via integration of a pretrained network with a MDN. By replacing the electromagnetic simulation with an external pretrained network that learns the correlation between optical performance and structure to evaluate the quality of the output structure, our proposed MPSN algorithm circumvents the problem of discarding potentially superior solutions. To identify the best structural parameters, we sample the mixture Gaussian distribution multiple times and input the results into the pretrained network for optical performance prediction.

As a demonstration, we use MPSN for the inverse design of metasurface-based structural colors^27–31^. Structural colors employ the scattering and interference of light from various structures to display a diverse range of colors^32–41^ and have been used in applications in fields such as high-resolution pattern design^42^, color switching^43^, 3D printing^44^, and device coloring^42^. Wide gamut color design represents an essential capability in structural color applications, enabling diverse color generation across the visible spectrum^45–48^. We select this problem to benchmark our capabilities in nanophotonic design, as achieving a precise color-to-structure relationship remains significantly challenging. The metamerism phenomenon^49^ posits that different spectra may display identical colors indistinguishable to the human eye, resulting in a highly one-to-many relationship from color to structure. We design a structure comprising a square ring and a square column. The reflectance of the structure is calculated using rigorous coupled wave analysis^50,51^ (RCWA), and the chromaticity coordinates are obtained using the color-science library in Python, which is used to build the dataset. After training, MPSN achieved a prediction accuracy up to 99.9% and a mean absolute error (MAE) of less than 0.002. MPSN not only outputs multiple results but also simplifies the attainment of optimal structures. We anticipate that MPSN will have practical applications in addressing non-uniqueness problems in the inverse design of nanophotonics. Our findings also offer valuable insights for addressing future inverse design challenges in plasmonic nanostructures^52^ and waveguides^53^ using deep learning techniques.

## Results

### Overview of our approach

**Fig. 1:**
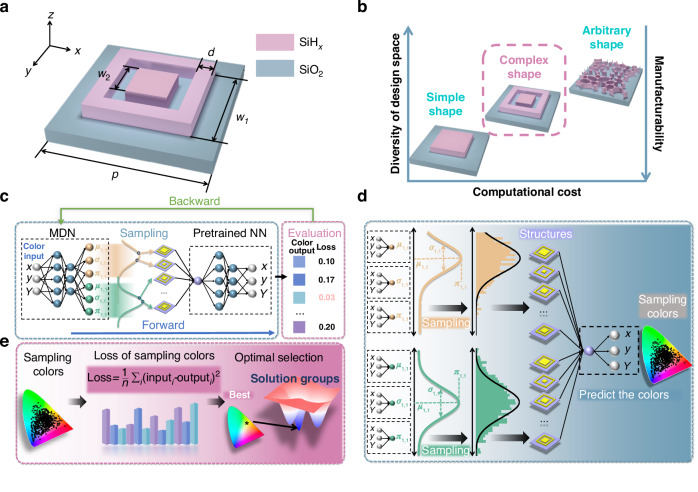
Schematic of the initial unit structure and MPSN computational pipeline. **a** Unit cell design comprising a square ring and central pillar controlled by periodicity $p$, ring width ${w}_{1}$, pillar width ${w}_{2}$, and ring gap $d$. **b** Tradeoff between the diversity of design space, manufacturability and computational cost for different structures. **c** Diagram of the MPSN architecture. MPSN consists of a mixture density network mapping from color to material distribution and a pretrained network mapping from material parameters to color. The two networks are connected by sampling the mixture Gaussian distribution multiple times. Select the optimal structure for training the network after evaluation of all sampling results. **d** Details of the Network. MDN outputs the mixture Gaussian distribution of the structure, sampling this distribution several times can yield many different structures. Then all structures are input into the pre-trained network to predict the colors. **e** Details of the evaluation step. The smallest MSE between sampling colors and real colors is selected for the network training

The initial unit structure is a key component in structural color design. Designing the initial shape requires ensuring polarization insensitivity, which necessitates symmetry in both the $x$ and $y$ directions. So, a metasurface that comprises a square ring and a square column is designed, as shown in Fig. 1a. The optical properties of the structure are controlled by four geometric parameters: periodicity $p$, ring width ${w}_{1}$, ring gap $d$, and pillar width ${w}_{2}$. The grating layer material is SiH_*x*_ which exhibits high refractive index and low absorption in the visible spectrum, with SiO_2_ serving as the substrate.

The unit cell configuration critically influences neural network design, dataset requirements, and ultimate training outcomes. Common structural variants include simple single-pillar designs, multi-pillar complexes, and high dimensional topology-controlled architectures as shown in Fig. 1b. Increasing geometric degrees of freedom proportionally elevates computational costs while reducing fabricability. The selected configuration demonstrates optimal balance, maintaining substantial design freedom while enhancing manufacturability and reducing computational costs to enable rapid large-scale dataset generation. An 8,411-group data set is created by randomly generating the structure parameters. More details about the design can be found in Supplementary Notes 1 and 2.

The MPSN comprises two key components: a color-to-structure MDN serving as the inverse design generator, and a pre-trained structure-to-color forward prediction network connected in series. This integrated configuration establishes an end-to-end color-to-color mapping framework that effectively resolves non-convergence issues stemming from the inherent non-unique structure-color relationships as shown in Fig. 1c. MPSN enables a larger spatial search by sampling the mixture Gaussian distribution of the output from the generative network to obtain multiple structures. The distribution can be mathematically expressed as:

1 $$P\left(\theta \right)=\mathop{\sum }\limits_{k=1}^{k}{\pi }_{k}{\mathcal{N}}\left(\theta |{\mu }_{k},{\sigma }_{k}\right)=\mathop{\sum }\limits_{k=1}^{k}{\pi }_{k}{e}^{-\frac{{\left({{x}}-{{{\mu }}}_{{{k}}}\right)}^{2}}{2{{{\sigma }}}_{{{k}}}^{2}}}$$

where ${\pi }_{k}$, ${\mu }_{k}$, and ${\sigma }_{k}$ denote the mixture weight, mean, and covariance matrix of the $k$-th Gaussian component, respectively.

MDN gives a mixture Gaussian distribution for structural parameters, while the pretrained network accepts only one structural parameter, which causes a mismatch between the number of outputs and inputs. The VAE faces the same problem, but it samples the potential distribution once and feeds results to the next network. With this approach, MPSN loses many results, and the ability of the network to find the best solution is reduced. Therefore, our strategy is to sample the mixture Gaussian distribution multiple times and pass all results into a pretrained network to predict colors, as shown in Fig. 1d. The sampling procedure is implemented via the reparameterization trick:

2 $${x}_{i}^{m}\left(\theta \right)=\mathop{\sum }\limits_{k=1}^{k}{z}_{k}^{m}\left[{\mu }_{k}+{\sigma }_{k}{\mathcal{N}}\left(0,I\right)\right]$$

where ${x}_{i}^{m}$ represents the result of the ${m}^{{th}}$ sampling for the ${i}^{{th}}$ structural parameter, ${\mathcal{N}}\left(0,I\right)$ represents standard normal distribution, and ${z}_{k}^{m}={\rm{Categorical}}({\pi }_{k})$, representing the categorical distribution^54^.

During sampling, mixture weights of all distributions are normalized, and the probability of selecting each distribution is determined based on the ${z}_{k}^{m}$. Sampled structural parameters are fed into the pre-trained network, predicting corresponding colors as

3 $${\hat{y}}_{i}^{m}\left(\theta \right)={g}_{\phi }\left({x}_{i}^{m}\right)$$

where ${g}_{\phi }$ and $\phi$ represent the function and the parameters of pre-trained network, respectively.

Then, we evaluate all structures by calculating the mean square error (MSE) with the real data, and select the best result with the smallest MSE for network training, as shown in Fig. 1e. The optimal structural selection methodology adheres to the following criterion:

4 $${\theta }_{i}^{* }={\mathrm{arg}}\mathop{\,\min }\limits_{\theta \in \varTheta }{\mathbb{E}}\left[{{||}{y}^{m}-{\hat{y}}^{m}\left(\theta \right){||}}^{2}\right]$$

where ${\theta }^{* }$ denotes the optimal structural parameters, ${y}^{m}$ represents the target color, ${\hat{y}}^{m}\left(\theta \right)$ is the predicted color, and $\varTheta$ defines the feasible parameter space.

The network training objective fundamentally minimizes the divergence between the output distribution $\hat{p}\left({y}^{m}\right)$ and the true data distribution $p\left({y}^{m}\right)$. Our enhanced sampling strategy achieves this by optimizing mixture parameters ${\pi }_{k},{\mu }_{k},{\sigma }_{k}$ to align the generated distribution ${g}_{\phi }({x}^{m})$ with target responses ${y}^{m}$, thereby ensuring $\hat{p}\left({y}^{m}\right)$ comprehensively covers high-probability regions of the empirical data distribution.

On the one hand, the MPSN utilizes end-to-end network properties to enhance the convergence. On the other hand, it expands the solution space by sampling a mixture Gaussian distribution. This distribution comprises several Gaussian distributions with varying weights, each of which represents a probability density profile of a singular solution. The normalized weight corresponds to the probability of that solution being sampled. When the inverse network outputs structural parameters instead of a mixture Gaussian distribution, some potential design solutions may be discarded, resulting in only one solution for a given design goal. This approach significantly limits output diversity and compromises network convergence. Without sampling the mixture Gaussian distribution, the two networks cannot be combined, and the pretrained network remains unusable. Consequently, training an inverse network of color-to-structure distributions is necessary, but the number of preset Gaussian distributions may not correspond to the number of actual simplex solutions, which could negatively impact the convergence of the network. To address this challenge, we sample the mixture Gaussian distribution multiple times. When the number of samples is too small, several Gaussian distributions may converge to the same solution. Conversely, an excessively large sample size can cause the model to retain only the best-performing distribution during training, discarding suboptimal ones and potentially leading to overfitting. We configure the number of Gaussian mixture components to no more than double the sample count, ensuring non-zero selection probabilities for all distribution elements. This strategic ratio maintains comprehensive parameter space exploration while maximizing solution diversity in the inverse design process.

We design both forward and inverse networks consisting of fully connected layers. The forward network employs four layers to facilitate the mapping from structure to color, while the inverse network, which utilizes 10 mixture Gaussian distributions, also employs four layers to achieve the mapping from colors to the structural parameters. It is important to note that individual networks are constructed for each distribution of $\mu$, $\sigma$ and $\pi$, as illustrated in Fig. 1d. This is done to address the issue of gradient instability and enhance network convergence. Training outcomes under different sampling frequencies are shown in Supplementary Note 3, revealing that both insufficient and excessive sampling iterations compromise network performance. The latter degrades the accuracy while the former reduces the solution diversity, highlighting the importance of balanced sampling parameter selection. The results demonstrate that setting the sampling count to 15 optimally balances the accuracy and diversity in our framework, achieving a robust prediction fidelity while expanding the range of valid structural solutions. We first train the forward network to achieve efficient and accurate color prediction instead of using the EM simulation. Then the weights and biases from the pretrained network are fixed to train the inverse network. As previously mentioned, the output of the inverse network is a mixture Gaussian distribution of structural parameters. We sample this distribution 15 times and pass the results into the forward network to obtain predicted colors. Then, the structural parameters with the smallest MSE against the actual color data are selected to train the inverse network. Details of the network are provided in Supplementary Note 3. Each non-unique solution in the dataset corresponds to a Gaussian component in the MPSN output. Redundant components are pruned by assigning near-zero weights to prevent sampling, while all Gaussian distributions remain mutually independent. Detailed computational results are also provided in Supplementary Note 3.

### Comparison of various algorithms

**Fig. 2:**
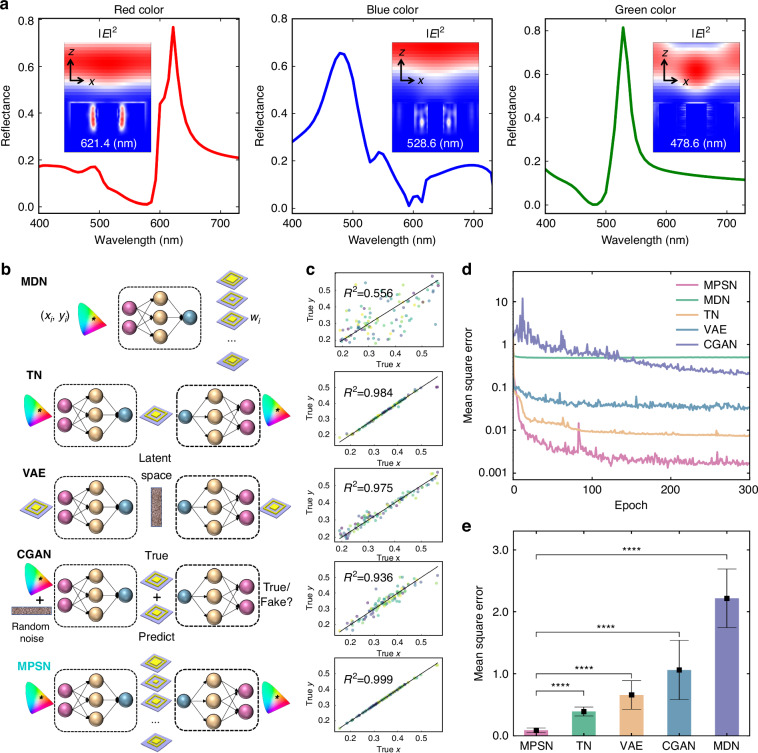
Comparative performance of MPSN. **a** Reflectance spectra for RGB primary colors designed by MPSN, with corresponding ${xz}$-plane electric field profiles at their characteristic reflection peaks**. b** Schematic structure of MDN, TN, VAE, CGAN and MPSN. **c** Prediction results on the test set, where the colored dots indicate the scatter plots of real data and predicted data, and the red line is the function $y=x$. **d** Convergence curves of the MSE (dB) of the test set during training for the five networks. **e** Histogram of the mean of the MSE of the five networks from the same 10 test sets, along with confidence intervals and confidence levels. The *p*-value is calculated by *T*-test and is less than 0.01%, which means that there is a significant difference between the two groups

We first implement MPSN to design RGB primary-color structures shown in Fig. 2a, achieving strong reflection peaks at 621.4 nm (red), 528.6 nm (green), and 478.6 nm (blue). Corresponding ${xz}$-plane electric field profiles at these wavelengths exhibit pronounced reflected fields with negligible transmission. Notably, strong electric field resonances emerge within ring-pillar gaps for both red-color and blue-color designs, while the green-color design exhibits no observable resonance yet generates strong reflected fields. To further demonstrate that MPSN improves the convergence and accuracy of the network, we train several typical networks and compare their training results, as shown in Fig. 2b–e. Schematic diagrams of the five neural network models and the test results after training are shown in Fig. 2b, c, respectively. The test results are represented by scatter plots of the chromaticity coordinates of the real data from the test set alongside the chromaticity coordinates of the predicted data. Colored dots represent the predicted chromaticity coordinates and the $y=x$ function is represented by the red line. ${R}^{2}$ is calculated as the square of the Pearson correlation coefficient. Higher ${R}^{2}$ values indicate better agreement between model predictions and the true data, reflecting the higher precision.

The accuracy of DNN that performs color-to-structure mapping is significantly affected by the non-uniqueness between the color and structure, making it unsuitable for use as an inverse design tool^19^. Since previous studies have analyzed the accuracy of DNNs in detail^19,20^, we focus on other NN modes here. MDN maps color coordinates to structure parameters using mixture Gaussian distributions^19^. Due to incorrect parameter selection, its design results can be even worse. TN operates in a color-to-color mapping, bypassing the non-unique challenge and further improving the convergence of the network^20^. As a result, its accuracy is significantly improved. However, the limitation of TN in generating multiple solutions, stemming from its single-output architecture, leads to unsatisfactory color predictions in certain scenarios. VAE is a classic end-to-end network primarily intended for unsupervised learning, but improvements in the network structure allow it to be a viable alternative for inverse design tasks^26^. This study proposes the critical role of the potential distribution in classifying the metasurface structure, optical properties, and predicting the metasurface structure itself. CGANs also incorporate a random noise to alleviate the non-uniqueness problem. By manipulating the noise, they can produce different outcomes, resulting in multiple solution outputs^21^. VAE and CGAN address one-to-many problems through stochastic sampling in latent spaces. However, because these spaces lack physical interpretations, their inherent randomness introduces quantifiable uncertainty. This contributes to lower accuracy compared to TN^24^. In contrast, MPSN outputs distributions with explicit physical meaning, each corresponds to a distinct potential solution (More detailed statistical analyses of MPSN, CGAN, and VAE outputs appear in Supplementary Note 4). As a result, the final prediction results of the MPSN demonstrate significant improvement over the other networks, as shown in Fig. 2c. It should be noted that the accuracy is calculated via the Pearson correlation coefficient, which reflects the network’s ability to fit the dataset and is independent of the accuracy of the RCWA method used to generate the data. To validate the reliability of the dataset, we include an analysis of the convergence behavior of RCWA and a comparative evaluation of its accuracy against finite difference time domain (FDTD) and finite element method (FEM) in Supplementary Note 5. All three methods demonstrate high accuracies, while RCWA offers faster computational speed, making it the preferred candidate for generating the dataset. Hyperparameter configurations for each benchmark network are shown in Supplementary Note 6.

Figure 2d presents a comparison of MSE convergence curves for the test set during the training process of the five networks. The vertical coordinate reflects the logarithmic value of MSE (dB), while the horizontal coordinate represents the number of training generations. Figure 2e displays the histogram of the MSE of 10 test sets, including the confidence interval and level of the results. The MSE can be obtained by $\frac{{\sum }_{i}^{N}{({\hat{x}}_{i}-{x}_{i})}^{2}}{N}$, where ${\hat{x}}_{i}$ represents the predicted color coordinate, ${x}_{i}$ represents the true color coordinate and $N$ represents the number of the test dataset. Notably, the convergence of DNN is hindered by the non-uniqueness problem, while the convergence of MDN is impacted by the parameter selection issue of the mixture Gaussian distribution. TN has improved network convergence due to its end-to-end design, but the accuracy suffers as a result of the single output result. VAE and CGAN demonstrate reduced output stability due to their inherent dependence on stochastic noise inputs. In contrast, MPSN not only yields a better convergence but also has the ability to output multiple results, resulting in a significant improvement in accuracy. To demonstrate the general applicability of MPSN, a complementary structure-to-spectrum network is implemented (see Supplementary Note 7), establishing bidirectional mapping capabilities between photonic structures and their optical responses within the same framework.

**Fig. 3:**
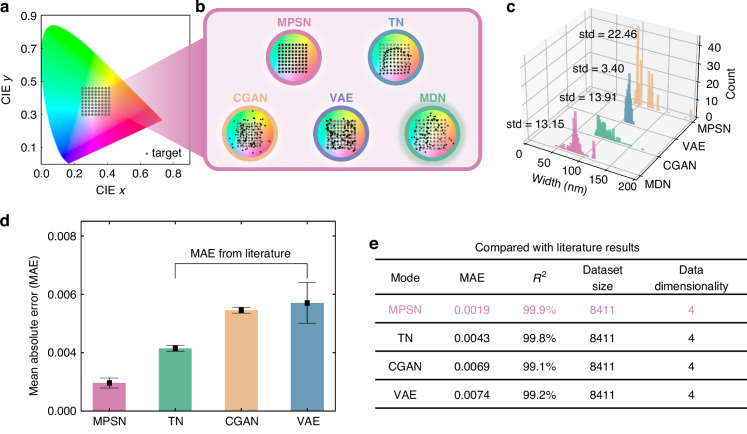
Accuracy comparison in structural color design. **a** Target colors on the CIE 1931 chromaticity diagram (gray dots). The resolution (minimum distance) of the colors is 0.02. **b** Design results of the five networks. We simulate and calculate colors corresponding to various structures designed using different networks and label them with black stars. **c** Diversity of four networks. Each network makes 200 predictions for the same color. Predictions of those networks are plotted as a statistical histogram. Standard deviation (std) quantifies solution diversity across all four networks, with results annotated beside on the left of the histogram. **d** MAE comparison of MPSN and literature results^24^. **e** Comparison of MAE and Pearson correlation coefficient for MPSN and literature results^24^. All comparisons are made using the datasets provided in the literature

Then we use each of the five networks to design a series of colors in the CIE 1931 chromaticity diagram, as shown in Fig. 3a–c. Figure 3a and b demonstrate an accuracy comparison of the design results, where the gray dots on the CIE 1931 chromaticity diagram are target colors. Figure 3b shows the design results of the neural networks. MPSN and TN can predict the structure and the corresponding color. To address potential discrepancies in color predictions using forward networks, a validation step through RCWA simulations is implemented to calculate reflectance spectra and derive corresponding color coordinates. These validated results are subsequently annotated with black stars in our visualizations. These results demonstrate that MPSN produces the most accurate color designs. The TN can correctly predict most colors, while MDN have limited accurate predictions. Since both MPSN and MDN can generate multiple possible structures, we sample 100 predictions from each network per color and select the result with the smallest Euclidean distance to the design target. In contrast, TN can only output one structure, which only needs to be designed once.

We then evaluate the diversity of the four networks, count the predictions for one of the colors above and plot the distributions of the pillar widths as histograms, as shown in Fig. 3c. Standard deviation (std) quantifies diversity for each dataset, with higher values indicating greater solution variation. These metrics for all four models are numerically annotated beside the histogram. MPSN, MDN, CGAN and VAE exhibit high output diversity, resulting in a structure size range of approximately 200 nm. TN cannot generate diverse structures, primarily due to its network architecture. This leads to the fact that although TN ensures network convergence, it does not yield optimal design results for any color, so the diversity of TN is not displaced here. MPSN converges the structure to two peaks, representing the most probable distribution of the design results. CGAN and VAE generate unimodal distributions in this case, indicating their inability to capture the multi-solution nature of the inverse design problem. TN achieves high-precision inverse design, but lacks solution diversity to generate multiple structures. MDN exhibits the highest diversity; however, due to a mismatch between the number of Gaussian distributions and the degeneracy present in the dataset, the distribution becomes overly dispersed, making it challenging for the network to identify the optimal solution.

All comparative evaluations utilize our proprietary dataset to train models from ref. ^23^, while we conversely employ the datasets from ref. ^23^ to train our MPSN. An accuracy comparison of structural color design performance between the proposed MPSN and established models including TN, CGAN, VAE is presented in Fig. 3d and e, with benchmark data drawn from published literatures^24^. Figure 3d displays an MAE comparison of the computed MPSN and the other three models. The MAE is computed by $\frac{{\sum }_{i}^{N}\left|{\hat{x}}_{i}-{x}_{i}\right|}{N}$, where ${\hat{x}}_{i}$ represents the predicted color coordinate, ${x}_{i}$ represent the true color coordinate and $N$ represent the number of the test dataset. Comparisons of ${R}^{2}$ and MAE are shown in Fig. 3e. While TN, VAE, and CGAN demonstrate high accuracy, this performance corresponds to metasurface comprising single-cylinder structures with relatively simple geometries and solution spaces. When applied to our more complex structures, these models show reduced accuracy. As shown in Fig. 3, TN achieves only 98.3% accuracy on our dataset. This accuracy level results in deviations from the desired target for certain color designs. Similarly, CGAN and VAE fail to achieve higher design accuracy. Practical applications often require complex structures to meet demanding requirements such as anomalous deflectors and beam splitters. MPSN maintains high accuracy for complex structural design problems, making it advantageous for addressing inverse design challenges in real-world applications.

### Experimental validation

**Fig. 4:**
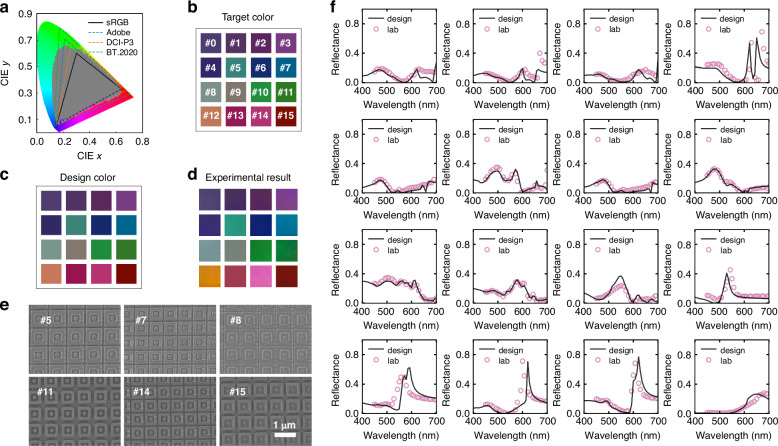
Experimental results of the color card sample. **a** Gray dots are the chromaticity coordinates of 1000 datasets, which cover most of the area of the Adobe RGB color gamut. **b** 16 target colors. **c** Design results of the 16 colors obtained using MPSN. **d** Experimental photograph of the color card for the 16 colors. **e** SEM images of the six labeled colors. Scale bar, 1 µm. **f** Reflectance spectra of the 16 colors in **d**

To experimentally evaluate the design capability of MPSN, 1000 samples are randomized within the dataset labelled on CIE 1931 to observe the color gamut of the structure (gray dots), as shown in Fig. 4a. A wide color gamut which covers 65.2% of the CIE color space can be observed. The MPSN provides a highly accurate design process for the inverse design, establishing a rapid and effective strategy for the design of structural color. As illustrated in Fig. 4b, the design target consists of 16 colors, which are selected to encompass the majority of the sRGB color gamut. Each of the 16 colors is designed and input into the trained design network. The network generates 100 structural predictions, which are then processed through the pre-trained model to derive corresponding color coordinates. The structure with the smallest MSE from the design target is chosen as the final design. Experimentally obtained colors are shown in Fig. 4d. Full information of the designed parameters, fabricated measurements, and fabrication tolerance analysis is provided in Supplementary Note 8. With the trained network, only 0.01 s is required to predict the corresponding structure. The total time spent is only 18.80 h (dataset generation: 18.79 h, forward network training: 35.18 s, inverse network training: 81.86 s). Detailed information about computing devices and the time consumed for each step is provided in Methods. In contrast, conventional optimization algorithms typically demand several days (detailed information is provided in Supplementary Note 9). This dramatic reduction in computational time enables efficient and precise structural color design solutions while maintaining performance parity with traditional methods. The scanning electron microscope images of 6 colors are shown in Fig. 4e. Reflectance spectra for 16 colors are experimentally recorded using a spectrometer as shown in Fig. 4f. Angular stability remains critical for structural color applications. We analyze reflectance spectra of Color 0, Color 11 and Color 14 in Fig. 4b at incident angles of 0°, 10°, 20°, 30°, and 40° in Supplementary Note 10.

**Fig. 5:**
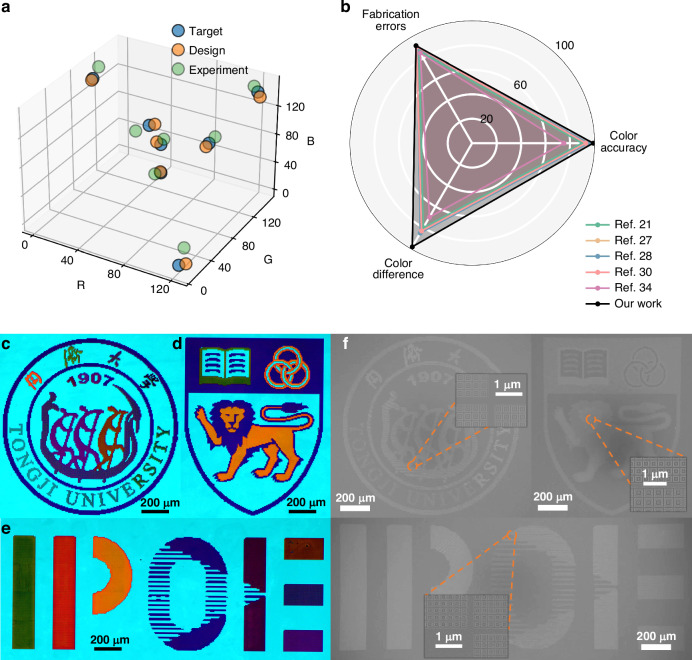
The microscopy images of the 3 painting samples. **a** RGB values for nine colors. Comparison of target, design, and experimental results. **b** Comprehensive comparison of color accuracy, color difference, and fabrication errors. Color accuracy is measured by the MAE between target and experimental values. Color difference represents the distance between two colors. Fabrication error denotes the deviation between target and experimental structures. Photographs of experimental observed logos of **c** Tongji University, **d** National University of Singapore, and **e** Institute of Precision Optical Engineering. **f** SEM images of the three samples. Scale bar, 200 µm. Insets show enlarged different color regions. Each pixel is designed with a size of 6.7 µm and filled with the same structure. Scale bar, 1 µm

As illustrated in color cards, the four monochromatic colors (red, orange, green and blue, corresponding to Nums. 14, 12, 11 and 7, respectively) exhibit a single reflective peak with a clear tendency of blue-shift. The data points exhibit a compact clustering pattern distal to the chromaticity boundaries within the CIE 1931 color space. Complex color schemes with precisely blended ratios are additionally engineered in the design framework. For instance, the purple color, designated as Num. 1, is comprised of red and blue colors. It is not solely reflective of red light, but also exhibits a high reflectivity in the blue band. This phenomenon can also be observed in other colors, which are composed of a combination of colors to create a more visually rich visualization. The detailed comparison of 9 colors is shown in Fig. 5a. RGB values are quantitatively compared across three sets: target colors, simulated colors, and experimentally realized colors. The RGB differences between target and designed colors are within 10, demonstrating the high accuracy of our network in inverse design. Comparative results for additional color variants are presented in Supplementary Fig. S17.

A comparative analysis between our approach and existing methodologies is presented in Fig. 5b, evaluating three critical metrics: color difference, color accuracy, and fabrication error. The color distance metric quantifies the minimum RGB distance between two designed colors using MAE, while color accuracy measures the MAE between experimentally captured colors and the simulated colors. Fabrication error is defined as the MAE between designed parameters and experimentally realized nanostructures, providing a quantitative assessment of manufacturing fidelity.

To show the versatility of MPSN in structural light design, three images are generated. Figure 5c–e display photographs of logos of Tongji University, National University of Singapore, and Institute of Precision Optical Engineering, respectively. The color of each pixel in these images is organized into columns and input into MPSN for 100 samples. The structure with the smallest MSE is selected as the final design. Although it is feasible to design a broader range of colors effectively, 16 colors from the color card are used as a demonstration to construct the entire image. Figure 5f shows scanning electron microscope images for Fig. 5c–e, highlighting representative regions from each image containing two-color structures. The single-pixel is designed with a size of 6.7 µm to ensure that all colors in the finite array case can be close to the infinite array. The selection method of single-pixel size and the complete experimental procedure are provided in Supplementary Notes 11 and 12.

## Discussion

In this work, we propose an end-to-end mixture probability sampling network by concatenating a pretrained network and a mixture density network. MPSN not only solves the non-uniqueness problem of structural color design but also enables the output of multiple solutions, ensuring a high probability in obtaining an optimal structure. The effectiveness of MPSN in identifying the ground truth of the testing set reaches 99.9%, which is superior to other neural networks used in metasurface inverse design. MPSN provides a mixture Gaussian distribution of structural parameters, giving users a considerable space to choose from. This greatly improves the design freedom. Our design achieves a target color using only a single cell, improving color resolution compared to those three-cell structures (where separate cells are dedicated to R, G, and B). Experimental results confirm that the network outperforms in structure-color inverse design.

Practical optical applications typically require more complex structures to achieve targeted functionalities. These complex structures possess more sophisticated solution spaces with pronounced one-to-many mappings. As shown in Fig. 3, TN, CGAN and VAE fail to achieve higher accuracy on our complex dataset. In contrast, our enhanced-sampling MPSN offers key advantages for such complex structures by modeling non-unique solutions through Gaussian mixture distributions. By leveraging multiple sampling iterations integrated with a pre-trained network, it further enhances accuracy, thereby contributing to solving inverse design problems in practical optical applications. In contrast to MDN networks, our network samples multiple instances from Gaussian mixture distributions, evaluates them via a pre-trained network, and uses optimal samples to train the primary model, with the accuracy influenced by the sampling frequency. Compared to VAE that employ probabilistic density estimation, our architecture achieves a comparable functionality with a simpler structure and greater diversity through explicit Gaussian mixture modeling. This probabilistic framework demonstrates significant advantages when processing noisy, multi-modal datasets, as it inherently learns probabilistic distributions rather than specific numerical values. This design enables flexible user-driven result selection through post-hoc filtering of the generated distribution space. Compared to traditional electromagnetic solvers like RCWA, FDTD, and FEM, MPSN substantially accelerates computation. Detailed comparative analyses are provided in Supplementary Note 13. In addition, Physics-Informed Neural Networks (PINNs)^55–60^, which integrates physical constraints, allows for more accurate solutions without heavy reliance on data. Implementing PINNs instead of pre-trained networks will reduce data reliance while enhancing prediction accuracy. This methodology inherently enforces physical plausibility constraints during the sampling process, effectively eliminating non-physical pseudo-solutions while maintaining solution diversity. Our work can also be extended to metasurface scattering prediction, optical efficiency optimization, and spectral design. With appropriately constructed datasets, MPSN also has strong potential for broader applications, including Dirac-like cone zero-index metamaterials^61,62^ and exceptional points in optical system^63^, using the same design methodology.

## Materials and methods

### Training data set generation

The metasurface is characterized by a periodic array structure, which is determined by four key parameters, namely, ${w}_{1},{w}_{2},{w}_{3},{w}_{4}$, as mentioned above. Additionally, the structure is surrounded by periodic boundaries, which led us to utilize the RCWA tools in the homemade software WGallop-v4.0^64,65^. To address the challenge of generating a significant amount of data within a limited timeframe, 50 points are sampled uniformly between wavelengths of 400 to 750 nm and select harmonic levels of [-10, 10] in both $x$ and $y$ directions. Convergence and accuracy analysis of RCWA are shown in Supplementary Note 5. The computation time per data point is approximately 8 s, leading to a total processing time of about 18.79 h for the complete dataset containing 8411 samples.

### Training process

MPSN is composed of two parts. The first part is a forward network that is responsible for mapping the metasurface structure to its corresponding color. This network comprises four fully connected layers with an input dimension of 4 and an output dimension of 3. Each layer uses the ReLU activation function. The second part of MPSN is an inverse network that maps the color to the metasurface structure. This network also consists of four fully connected layers, with an input dimension of 3 and an output of *N* mixture Gaussian distributed parameters. To ensure optimal performance, each parameter is mapped by a separate network, as outlined in Supplementary Note 3. The loss function employed in the training process is the MSE, and the learning rate is initially set to 0.001. As the number of training generations increases, the learning rate is multiplied by 0.8 every 100 generations. The entire training process is executed on a workstation equipped with an Intel Core i7-10750H CPU, 64-GB RAM, and an NVIDIA GeForce RTX 3060 GPU. The pre-trained network demonstrates a runtime of 35.18 s with 1.2-MB memory allocation, while MPSN training requires 81.86 s and utilizes 1.3-MB memory resources. Both networks employed identical training configurations using 80% of the data for training, a batch size of 256, and 300 training epochs.

### Model evaluation

The network generates structural parameters by inverse network and the corresponding predicted colors when given a target color. A critical limitation arises as inverse networks may generate non-physical pseudo-solutions that pre-trained networks fail to detect. The pre-trained network may still assign them realistic-looking color predictions similar to actual data, fundamentally undermining the reliability of pre-trained models for solution validation. In the work, RCWA is used to compute the actual colors from the proposed structures. Accuracy is evaluated in two ways: first, by comparing predicted colors with ground-truth values using average error measurements, and second, by testing the network’s ability to handle user-specified colors outside the dataset. For diversity assessment, we repeatedly generate 200 designs for the same target color and analyze the parameter dispersion in the solution space.

### Model comparison

Our algorithm comparison involves two key evaluations. First, we tested existing models (TN, CGAN, and VAE) on our dataset by adjusting parameters from their original code implementations, with results shown in Fig. 2. Second, we trained our MPSN model using datasets from published studies, with comparative outcomes displayed in Fig. 3. These dual comparisons demonstrate MPSN’s unique strength in solving “one-to-many” challenges in structural color inverse design. The method not only achieves higher prediction accuracy but also explores a broader range of valid solutions compared to conventional approaches. TN, CGAN, and VAE models are evaluated on identical computational hardware to MPSN, with all models assessed independently under the same conditions. All models utilize identical pre-trained networks, datasets, and data partitioning strategies. Training runtimes and memory footprints are quantified as follows: the TN model completes training in 84.70 s with 1.2-MB memory allocation, while VAE training requires 156.66 s and 2.5-MB memory. CGAN implementation demands 172.80 s and 567-KB memory resources. Comparative computational complexity assessments for the four models are described in Supplementary Note 14.

### Fabrication and measurements

SiHx metasurfaces are fabricated with the electron beam lithography technique, followed by an inductively coupled plasma reactive ion etch process. Basically, the inversed nano-pattern is generated within a ZEP electron beam resist. The metasurface pattern is written using a 100 keV electron beam lithography (EBPG5200, Raith) system. Then SiH_*x*_ nanostructures are produced by the ICP-RIE etching (see details in Supplementary Note 11). Samples are placed onto an optical microscope with a PHILIPS projection lamp type 7388 light source to visualize the images. The reflection spectrum is recorded with an angle-resolved microspectrometer (ARMS) under the ×100 objective lens.

## Supplementary information

Supplemental Material

## Data Availability

All data needed to evaluate the conclusions in the paper are present in the main text and the supplemental information. The datasets generated and analyzed during this study are available on GitHub at: https://github.com/Breathay/colortrain.git.

## Code availability

The source code is freely available and can be found on GitHub at: https://github.com/Breathay/colortrain.git.
