# Supplementary material for: Ultraprecision, high-capacity, and wide-gamut structural colors enabled by a mixture probability sampling network
Source: Light Sci Appl. 2026 Mar 11;15:164. doi: 10.1038/s41377-025-02122-3 (PMC12976068; doi:10.1038/s41377-025-02122-3)
Supplement: Supplementary file 1 — Supplemental Material [file 41377_2025_2122_MOESM1_ESM.pdf]

Supplementary Information for

Ultraprecision, high-capacity, and wide-gamut structural colors enabled by a mixture probability sampling network

Zeyong Wei[1,2,3,4,5,§], Weijie Xu[1,2,3,4,5,§], Siyu Dong[1,2,3,4,5,§], Xiaojia Liang[1,2,3,4,5], Jingyuan Zhu[1,2,3,4,5], Hui Zhang[1,2,3,4,5], Kaixuan Li[6], Lei Jin[7], Zhanshan Wang[1,2,3,4,5], Yuzhi Shi[1,2,3,4,5,8*], Gang Yan[1,3,*], Cheng-Wei Qiu[6,*] and Xinbin Cheng[1,2,3,4,5,*]

[1]School of Physics Science and Engineering, Tongji University, Shanghai 200092, China

[2]Institute of Precision Optical Engineering, School of Physics Science and Engineering, Tongji University, Shanghai 200092, China

[3]MOE Key Laboratory of Advanced Micro-Structured Materials, Shanghai 200092, China

[4]Shanghai Institute of Intelligent Science and Technology, Tongji University, Shanghai 200092, China

[5]Shanghai Frontiers Science Center of Digital Optics, Shanghai 200092, China

[6]Department of Electrical and Computer Engineering, National University of Singapore, Singapore 117583, Singapore

[7]School of Electronics and Information Engineering, Hangzhou Dianzi University, Hangzhou 310018, China

[8]Shanghai Eye Diseases Prevention & Treatment Center, Shanghai Eye Hospital, Shanghai 200092, China

[§]The authors contributed equally to this work

*Corresponding authors: yzshi@tongji.edu.cn (Y.S.); gyan@tongji.edu.cn (G.Y.); chengwei.qiu@nus.edu.sg (C.-W.Q.); chengxb@tongji.edu.cn (X.C.)

Supplementary Text

Supplementary Note 1: Unit structure design

The initial unit structure is a key component in structural color design. Currently, material selection predominantly focuses on all dielectric materials with high refractive index, such as Si and TiO_2. SiH_x exhibits a refractive index similar to Si in the visible light range, with even lower absorption under our coating process. Higher refractive index materials lead to more distinct resonances, which can expand the color gamut exhibited by the structure. Designing the structural shape requires ensuring polarization insensitivity, which necessitates symmetry in both the x and y directions, such as square, circular, or ring-like structures. Since greater structural freedom enhances dispersion control, we introduce a square ring around the square pillar as shown in Fig. S1. This modification increases the degrees of freedom while minimizing fabrication complexity. Compared to Fabry-Pérot cavity, although the color gamut of the nanostructure is narrower, it provides enhanced on-chip integration and control capabilities.

We compute electromagnetic field distributions at the reflection peaks for the corresponding red and blue structures, as shown in Figs. S1b-S1d. Figure S1b shows the refractive index distribution of the cross-section of the structure. Figure S1c shows the electromagnetic field distribution for the red structure at 621.4 nm. Strong electric field resonance occurs between the square ring and square pillar, while strong magnetic field resonance is observed within the square pillar. Figure S1d shows the electromagnetic field distribution for the blue structure at 478.6 nm. Both the electric and magnetic fields exhibit strong resonance between the square ring and pillar.

Supplementary Note 2: Dataset details

Data set is a fairly important element in deep learning. A high-quality data set often makes a guarantee on the training results of neural networks. In this work, we investigate the metasurface features of dominant colors and design a fully dielectric 2D hydrogenated silicon grating (similar to silicon grating which is widely used for structural color due to its high refractive index, strong Mie resonance, and simplicity of fabrication). It is periodically aligned in both the x and y directions, the grating layer is hydrogenated silicon, the substrate layer is SiO_2, as shown in Fig. S2a. The material is encoded by the parameters w_1, w_2, w_3, w_4. The statistical histograms of the

parameters are shown in the Fig. S2b to demonstrate the homogeneity of our data set.The histogram of color statistics corresponding to these structures is shown in Fig. S2c. The grating height h is fixed at 100 nm. We randomly generate 8000 different sets of structural data and calculate the reflectance for positive incidence conditions using rigorous coupled wave analysis. In order to address the challenge of generating a significant amount of data within a limited time frame, 50 points are sampled uniformly between the wavelengths of 400 to 750 nm and select harmonic levels of [-8, 8] in both the x and y directions.

The tristimulus values in the CIE1931 system can be calculated using Equation (S1) based on the reflectance data:

$$
\begin{aligned}
X &= \int_{\lambda} R(\lambda)S(\lambda)\bar{x}(\lambda)d\lambda \\
Y &= \int_{\lambda} R(\lambda)S(\lambda)\bar{y}(\lambda)d\lambda \\
Z &= \int_{\lambda} R(\lambda)S(\lambda)\bar{z}(\lambda)d\lambda
\end{aligned}
\tag{S1}
$$

where $R(\lambda)$ represents the reflectance, $S(\lambda)$ represents the spectral power distribution of the light source, and $\bar{x}, \bar{y}, \bar{z}$ are the color-matching functions. The value of k is taken when Y is normalized to 100. Normalizing x and y yields the chromaticity coordinates:

$$
\begin{aligned}
x &= \frac{X}{X+Y+Z} \\
y &= \frac{Y}{X+Y+Z}
\end{aligned}
\tag{S2}
$$

Data preprocessing is a crucial step in deep learning. Since the numerical values of data may vary across different dimensions, the dimension with a larger magnitude holds greater significance. To avoid the interference caused by order of magnitude, the data set is normalized through subtraction of the average value and division by the standard deviation. In this work, 8,000 data sets are computed and divided into two categories: 80% of which are allocated to the training set for the network to learn knowledge, and 20% are reserved for the test set to evaluate the performance of the network. The test set results serve as the benchmark for evaluating the training results of the network. Poor results in the test set accompanied by good results in the training set may indicate over-fitting, suggesting the network model may be too complex. Conversely, poor results in both sets may indicate an unreasonable network design or issues with the data set, which are key concerns that must be addressed in deep learning.

Learning rate is also one of the important factors that affect the final training results of the network. A large learning rate decreases the accuracy of iterations, thus making it difficult for the network to find the optimal solution and repeatedly skipping the optimal solution. At the same time, a small learning rate will undoubtedly increase the computational cost. Therefore, in this paper, we vary the learning rate of the network according to different training periods, setting a faster learning rate at the beginning of training, and then slowing down the learning rate to allow the network to iterate with higher accuracy and find the optimal solution after gradually converging. The initial learning rate is set to 0.001. The learning rate is systematically reduced to 80% of its initial value following every 100 training iterations.

Supplementary Note 3: Network training

Training details

This section provides additional details on mixture probability sampling network training. MPSN is composed of two parts. The first part is a forward network that is responsible for mapping the metasurface structure to its corresponding color. This network is comprised of four fully connected layers with an input dimension of 4 and an output dimension of 3 as shown in Fig. S3. Each layer uses the rectified linear unit (ReLU) activation function. The second part of MPSN is an inverse network that maps the color to the metasurface structure. This network also consists of four fully connected layers, with an input dimension of 3 and an output of N mixture Gaussian distributed parameters. To ensure optimal performance, each parameter is mapped by a separate network.

The loss function employed in the training process is mean squared error (MSE), and the learning rate is initially set to 0.001. As the number of training generations increases, the learning rate is multiplied by 0.8 every 100 generations. Adam is chosen as the optimizer. The entire training process is carried out on a computer with an Intel Core i7-10750H CPU, 64-GB RAM, and an NVIDIA GeForce RTX 3060 GPU.

We first train the forward network to map structure to color. The 8411 sets of data are divided into 80% training sets, which are used to update the network parameters, and 20% test sets, which are used to evaluate network performance after each training cycle. We employ a batch training strategy, in which the network is fed with 256 sets of data at a time. Once the training on a batch is completed, the next 256 sets are fed into the network. This process is

repeated until all training sets have been processed, marking the end of one training cycle. The advantages of batch training include: (1) faster training speed, (2) reduced variance in parameter updates leading to improved network convergence, and (3) increased network generalization ability.

The convergence curve of the loss function during training of the forward network is displayed in Fig. S4a. Due to the adoption of batch training, the network converges rapidly with MSE dropping below 0.008 by the second cycle. The predictions of the forward network on the 20% test set are shown in Figs. S4b-S4d. The black dots represent the true values, while the predicted values are on the y-axis. The red line represents the line of equality $y = x$, and the R^2 value indicates the correlation coefficient, with values closer to 1 indicating more accurate predictions. As structure-to-color mapping is a strictly one-to-one relationship, even the simplest network can produce good training results, with R^2 values above 99.5%. This provides a basic guarantee for the subsequent training of MPSN in our work.

We then fix the parameters of the forward network and iterate the inverse network MDN for color-to-structure mapping. The output of MDN is a mixture of Gaussian distributions for structure, conventionally handled by maximum likelihood function. However, as MPSN requires the connection between MDN and the pre-trained forward network, we chose to randomly sample the mixture of Gaussian distributions 15 times. These 15 samples are then fed into the forward network to obtain color prediction values, which are compared with the true values. The result with the smallest MSE is selected to calculate the gradient and update the network parameters. The training results of the inverse network are shown in Fig. S5. Figure S5a displays the convergence curve of MSE on the test set during each training cycle, while Figures S5b-S5d show the results of chromaticity coordinate prediction. The prediction accuracy of the inverse network still maintains above up to 99.9%. This confirms the effectiveness of our network in solving the problem of network non-convergence caused by one-to-many relationships.

To demonstrate the Gaussian mixture properties of MPSN outputs, we input a random color $x = 0.31$, $y = 0.39$, $Y = 5.23$ for prediction and extract corresponding π, μ, and σ parameters. Ten Gaussian components per structural parameter m, d, t, w_2 are shown in Fig. S6. Each component's μ represents the parameter mean, π denotes selection probability, and σ indicates dispersion. Training results reveal minimal σ values, confirming that the network identifies non-unique solutions and precisely fits them with distributions. Redundant components show no

coincidence with existing distributions and are pruned by setting $\pi = 0$ during training, ensuring their exclusion from sampling.

We then compute the Pearson correlation coefficient matrix for these ten Gaussian components as shown in Fig. S7. Most components exhibit low correlations, confirming mutual independence between distributions. For parameter w, components 2 and 4 show high correlation due to their extremely low π weights. Similarly, elevated correlations in parameter w_2 result from negligible probability density at components with near-zero π weights.

Selection of the sampling number

We achieve an end-to-end design by repeatedly sampling from MDN and feeding the results into the pre-trained network. The number of samples significantly affects the outcome. Few samples may prevent the network from identifying the optimal solution, while an excessive number of samples may lead the network to overlook several degenerate solutions. Figure S8 illustrates the relationship between the number of samples and the final training results.

Figure S8a illustrates the relationship between the diversity of the MPSN and the sampling number. For single sampling (Number = 1), the network exhibits relatively low diversity but high accuracy, as shown in Fig. S8b. The output distribution gradually converges to a single solution, which is equivalent to directly using TN. When Number = 5, the network achieves the highest diversity, but the accuracy decreases, as shown in Fig. S8c. In this case, it is often challenging to identify the optimal solution, which corresponds to directly utilizing MDN. When Number = 10, the output distribution gradually converges to two degenerate solutions, representing the two most likely structural parameters. This increases diversity while maintaining accuracy. When the number of samples exceeds or equals 20, the network can sample almost every point in the distribution. This causes the network to focus only on the current optimal solution during evaluation and neglecting suboptimal solutions. As a result, the output distribution still converges to a single solution, which is comparable to the effect of directly using TN.

Supplementary Note 4: Statistical Analysis of MPSN, CGAN, and VAE Models

This section presents statistical analyses of MPSN, CGAN, and VAE, with all models incorporating stochastic processes. For a fixed chromaticity coordinate input ($x = 0.31$, $y = 0.39$),

each model performs 200 independent predictions as shown in Figs. S9 and S10. Figure S9a shows structural parameter *d* predictions. MPSN drives σ toward minimal values during training to precisely match target solutions. Although CGAN and VAE exhibit similar prediction expectations to MPSN, their single-sampling training mechanism produces substantially higher variance. Figures S9b and S9c present the predicted chromaticity coordinates x and y, respectively. Consistent with structural predictions, MPSN outputs cluster tightly around target values, while CGAN and VAE exhibit substantially greater dispersion due to higher uncertainty.

We present a bar plot of the predicted colors from each model as shown in Fig. S10. Expected values are annotated on the bars, with 95% confidence intervals shown. Results demonstrate that while MPSN incorporates stochastic processes, its cascading pre-trained network significantly reduces uncertainty through selective filtering. CGAN and VAE exhibit greater instability due to higher stochasticity.

Supplementary Note 5: Convergence and accuracy analysis of RCWA

This section demonstrates the convergence and accuracy of the RCWA method used in this study. Taking the No. 10 structure (green color) in Fig. 4b as an example, whose geometric parameters are listed in Supplementary Note 8 (Table S1). We compute the convergence behavior at a wavelength of 550 nm, as shown in Fig. S11. The Fourier orders are incrementally increased from [0, 0] to [20, 20]. The reflectance stabilizes at [9, 9] orders. For safe, we select [10, 10] as the parameter set for dataset generation, achieving an optimal balance between accuracy and computational efficiency. Then we compare the reflectance of RCWA, FDTD (Lumerical 2020 R2), and FEM (COMSOL 6.2) across the 400–750 nm wavelength range in Fig. S12. The three algorithms demonstrate nearly identical results, confirming the accuracy of the RCWA method.

Supplementary Note 6: Hyperparameter Configurations for Other Networks

For fair model comparison, VAE, TN, MDN, and CGAN use identical datasets and pre-trained network. All models maintain maximally similar network configurations. Both TN and MDN adopt three hidden layers with 128, 256, and 200 nodes. TN maps 200 nodes directly to four output parameters. MDN mirrors MPSN's architecture using three separate networks (200 input nodes, 4 output nodes) for mixture Gaussian distributions. VAE and CGAN implement

structures[1] as described in Ref. 1. Discriminator of CGAN contains four hidden layers with 64 nodes each, while generator uses three hidden layers with 64 nodes each. VAE employs three hidden layers with 64 nodes for both encoder and decoder. All models utilize the Adam optimizer with ReLU activation functions. All models use an initial learning rate of 0.001 with a decay multiplier of 0.8 applied every 100 epochs.

Supplementary Note 7: Spectral inverse design

To further validate the accuracy of MPSN, we extend the inverse design to spectral properties using the same dataset as in color inverse design. The network input is modified from (x, y, Y) color coordinates to reflectance values sampled at 50 equally spaced wavelengths across 400-750 nm. The dataset retains the original dimensions of (8000, 50) for inputs and (8000, 4) for outputs, matching the color inverse design configuration. The network architecture and training parameters are kept identical. The training results of the pretrained network are presented in Fig. S1, where Figure S13a shows the convergence curve of the test set loss function during training, and Figures S13b-S13c illustrate the spectral prediction results for three selected test cases.

The training results of the inverse design network are presented in Fig. S14. Figure S14a displays the convergence curve of the test set loss function during training, while Figures S14b-S14c illustrate spectral prediction results for three representative test cases. The use of MSE as the loss function drives the network to minimize the average error across the entire spectrum. This global optimization approach can result in underestimated reflectance peaks, where predicted values are systematically lower than the true values. This behavior provides one plausible explanation for the reduced reflectance observed in the final inverse designed structures.

Supplementary Note 8: Fabrication Errors and Tolerance Analysis

Table S1 lists designed and fabricated dimensions for the 16 structures in Fig. 4b, including parameters m, d, t, w_2. Primed superscripts (m', d', t', w_2') denote SEM-measured fabricated dimensions, all in nanometers. The values in parentheses represent the absolute error from the design target. The mean error between fabricated and designed structures is 6.02 nm, with a maximum error of 18.44 nm.

Table S1. Fabrication accuracy assessment.

m	d	t	w_2	m'	d'	t'	w_2'
67	90	114	312	68.62 (1.62)	94.01 (4.01)	108.29 (5.71)	313.25 (1.25)
64	78	130	276	63.87 (0.13)	69.07 (8.93)	129.46 (0.54)	278.43 (2.43)
74	80	142	308	80.37 (6.37)	76.13 (3.87)	133.45 (8.55)	305.79 (2.21)
25	60	40	148	19.90 (5.10)	70.42 (10.42)	33.68 (6.32)	147.00 (1.00)
69	85	102	110	71.14 (2.14)	86.09 (1.09)	93.29 (8.71)	111.43 (1.43)
33	136	66	106	33.81 (0.81)	129.60 (6.40)	59.17 (6.83)	118.30 (12.30)
59	81	123	114	64.59 (5.59)	82.07 (1.07)	122.20 (0.80)	106.98 (7.02)
33	100	56	122	29.18 (3.82)	92.17 (7.83)	49.16 (6.84)	132.10 (10.10)
20	269	56	228	12.29 (7.71)	279.60 (10.60)	46.09 (9.91)	239.60 (11.60)
29	250	171	180	27.50 (1.50)	242.00 (8.00)	170.50 (0.50)	192.50 (12.50)
73	208	166	144	82.61 (9.61)	210.67 (2.67)	168.65 (2.65)	136.88 (7.12)
46	41	56	60	39.66 (6.34)	52.87 (11.87)	43.43 (12.57)	66.09 (6.09)
25	60	69	72	22.66 (2.34)	54.76 (5.24)	60.42 (8.58)	83.08 (11.08)
44	57	88	42	36.03 (7.97)	60.44 (3.44)	72.07 (15.93)	60.44 (18.44)
28	70	60	86	25.76 (2.24)	70.51 (0.51)	48.82 (11.18)	94.92 (8.92)
183	83	102	100	191.57 (8.57)	86.44 (3.44)	98.72 (3.28)	101.68 (1.68)

Fabrication tolerance analysis for Structure 10 (green, Fig. 4b) proceeds as follows: First, correlation coefficients between structural parameters m, d, t, w_2 and color coordinates X, Y, Z are calculated across the dataset, with the correlation matrix visualized as a heatmap in Fig. S15a. Parameters m, d, t exhibit the strongest correlations, while w_2 shows weaker dependence. Next, ±20 nm perturbations are applied to each parameter, and the resulting mean RGB errors relative to target colors are plotted in Figs. S15b-S15d. The RGB error is calculated as $\frac{|R-\hat{R}|+|B-\hat{B}|+|G-\hat{G}|}{3}$, where R, G, B represent the error-free colors and $\hat{R}, \hat{G}, \hat{B}$ represent the colors with error consideration. All errors remain below 0.07, with curve colors representing actual spectral responses under each deviation. Finally, randomized ±20 nm errors are simultaneously applied to all four parameters, and the statistical distribution of color errors is histogrammed in Fig. S15d. Results confirm the robust fabrication tolerance that meets practical application standards.

Supplementary Note 9: Computation time analysis of traditional optimization algorithm

This section quantifies the computational execution times for genetic algorithms[2] (GA) and particle swarm optimization[3] (PSO). The GA follows methodology in Ref. 1, while PSO implements the framework in Ref. 2. Electromagnetic (EM) simulations constitute the primary computational bottleneck during iterative optimization procedures. Consequently, the total runtime for any optimization objective is determined by multiplying the number of simulations n by the duration per simulation t_i. Utilizing our rigorous coupled-wave analysis (RCWA) solver, a single-node computation across 50 wavelength points requires approximately $t_i = 8$ seconds (10×10 Fourier orders) on our computational device (Intel Core i7-10750H CPU, 64-GB RAM).

Computational time comparisons are detailed in Table S2. Based on established literature, we adopt a population size of $N_p = 50$ and uniform total iterations $T = 150$. For GA, the crossover count N_c and mutation count N_m determine simulation requirements. Each crossover necessitates two EM simulations, while each mutation requires one EM simulation. Thus, total simulations compute as $N_p + T(2N_c + N_m)$. For PSO, each particle requires one simulation per iteration, yielding total computations of $N_g + N_g \cdot T$. For N distinct optimization objectives, the total simulation count must be multiplied by N. Here we set $N_c = 15$, $N_m = 20$, $N = 16$ for the colormap design.

Table S2. Computational time comparisons of GA, PSO and MPSN.

Modal	Simulation times	Total time
GA	120800	268.4 hours
PSO	120800	268.4 hours
MPSN	8411	18.8 hours

Supplementary Note 10: Angular Stability Analysis

This section analyzes angular effects on Color 0, Color 11 and Color 14 from Fig. 4b. Figure S16 display spectra of Color 0, Color 11 and Color 14 at incident angles from 0° to 40°. Increasing incidence angles enhance long-wave reflectance and induce spectral redshift, yet both colors maintain minimal angular sensitivity, exhibiting negligible color shifts even at 40° incidence.

Supplementary Note 11: Fabrication details

The metasurface is fabricated on a glass substrate that is 1 mm thick. Firstly, a layer of SiH_x with a thickness of 100 nm is deposited on the glass surface via magnetron sputtering deposition system. Subsequently, the samples undergo standard electron beam lithography and dry etching manufacturing techniques to complete the fabrication process. The key steps are illustrated in Fig. S17.

Deposition of a SiH_x film on the glass substrate: An SiH_x film is deposited on the glass substrate using a magnetron sputtering deposition system (NSC-15, Optorun). The chamber is vacuum pumped down to 5.0×10^{-4} Pa at 25 °C. In this process, Si is the starting material and H_2 is injected to generate SiH_x. The deposition rate under these conditions is 0.25 nm s^{-1} for SiH_x. The thickness of the deposited SiH_x is 100 nm.

Photoresist spin-coating on wafer: ZEP520 electron beam photoresist is spin-coated with a thickness of approximately 80 nm. The piece is baked at 180 °C for 5 minutes.

Electron-beam lithography exposure and development: The metasurface pattern is written using a 100-keV electron beam lithography (EBPG5200, Raith) system. The beam current is 3 nA and the exposure dose is 220 μC cm^{-2}. Subsequently, the wafer is developed in pentyl acetate for 1 minute at room temperature.

ICP-RIE etching: The wafer is etched with a mixture of SF6 and CHF3 by Oxford PlasmaPro 100 Cobra300. The flow rates of the SF6 and CHF3 gases, working pressure, ICP power and temperature are maintained at 20 and 60 sccm, 10 mTorr, 1200 W and 5 °C, respectively. The etching is approximately 5 nm s^{-1}.

Removal of the residual photoresist: The etched sample is placed in a Remover PG solution and soak for 2 hours to remove the residual photoresist.

Supplementary Note 12: Selection of the single pixel size

Image resolution is a critical aspect of structural color design. We calculate the color for arrays composed of different numbers of structures, as shown in Fig. S18. We calculate structures with array sizes ranging from 3×3 to 11×11. To simulate the color of a single pixel, each structure is assigned Perfectly Matched Layer (PML) boundaries in both the x and y directions. Figure S18a shows the reflectance results. As the array size increases, the reflectance

spectrum becomes more similar to that of a periodic structure. Figure S18b shows the corresponding color results. For the 3×3 array, the color differs significantly from the periodic structure, while the 9×9 array is close to the periodic structure, and the 11×11 array almost matches it. Therefore, in image design, the pixel size is set to 6.7 μm to ensure that the color of each structure closely matches the periodic structure.

Supplementary Note 13: Computation time comparison of traditional solvers and MPSN

This section compares computation times of traditional EM solvers (RCWA, FDTD, FEM) and pre-trained networks, as detailed in Table S3. These algorithms achieve rapid computation: RCWA calculates diffraction efficiencies for periodic structures in 8 seconds (10×10 Fourier orders and 50 wavelength sampling points), while full-wave 3D solvers FDTD and FEM require about 2 minutes on our computational device (Intel Core i7-10750H CPU, 64-GB RAM). Nevertheless, integrating EM simulations with deep learning remains an active research area. The primary approach uses simulators as validators for deep learning models, such as replacing pre-trained networks in MPSN to enable realistic structural predictions. Alternatively, deep integration of automatic EM solvers within neural network frameworks shows promise. Both methods require calling EM solvers during every training epoch. Using the fastest RCWA algorithm under our framework (300 epochs, 80% training set ratio), complete training consumes 8411 samples × 0.8 × 8 = 14.95 hours per epoch. This is significantly slower than the pretrained network's 35.18 seconds inference time.

Table S3. Computational time comparisons of traditional solvers and pretrained networks.

Modal	Computation time per run	Total training time (300 epochs)
RCWA	8 s	4486 hours
FDTD (Lumerical)	122 s	68402 hours
FEM (CST Studio)	110 s	61674 hours
Pre-trained network	0.1 s	35.18 seconds

Supplementary Note 14: Computational Complexity Assessment

We evaluate the computational complexity of MPSN, CGAN, TN, and VAE models, quantifying model parameters, training multiply–accumulate (MACs), evaluation MACs, and

memory footprint which includes model parameters (Params), Activation functions, and gradients (Activations) during training.

Table S4. Computational complexity comparisons.

Model	Parameters	Training MACs/batch	Evaluation MACs/batch	Memory (Params)	Memory (Activations)
cGAN	22,405	151,488	146,752	~89.6-KB	~567-KB
MPSN	45,018	2,418,176	2,130,432	~177.7-KB	~1.3-MB
VAE	28,252	380,928	126,976	~0.49-MB	~2.5-MB
TN	85,612	553,056	184,352	~400-KB	~1.2-MB

References

1. Ma, T., Tobah, M., Wang, H. & Guo, L.J. Benchmarking deep learning-based models on nanophotonic inverse design problems. *Opto-Electron. Sci.* **1**, 210012 (2022).
2. Berto, P. et al. Tunable and free-form planar optics. *Nat. Photon.* **13**, 649-656 (2019).
3. Nugroho, F.A.A. et al. Inverse designed plasmonic metasurface with parts per billion optical hydrogen detection. *Nat. Commun.* **13**, 5737 (2022).

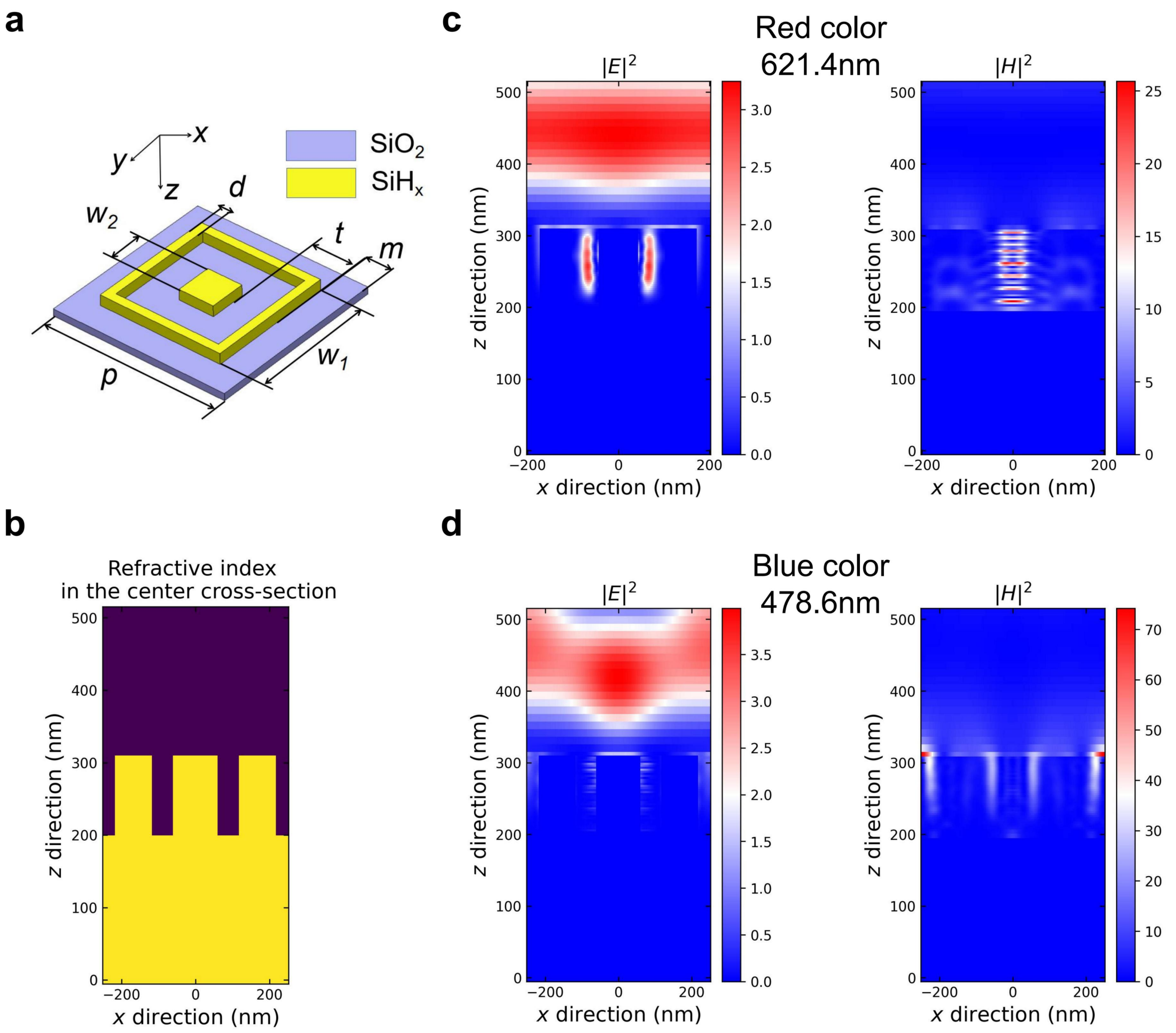

Fig. S1. Schematic diagram of unit structure and the field distribution calculations. a, Schematic of the unit structure. **b**, Refractive index distribution in the center cross-section. **c**, Computations of the electric and magnetic fields at 621.4 nm for the red structure. **d**, Computations of the electric and magnetic fields at 478.6 nm for the blue structure.

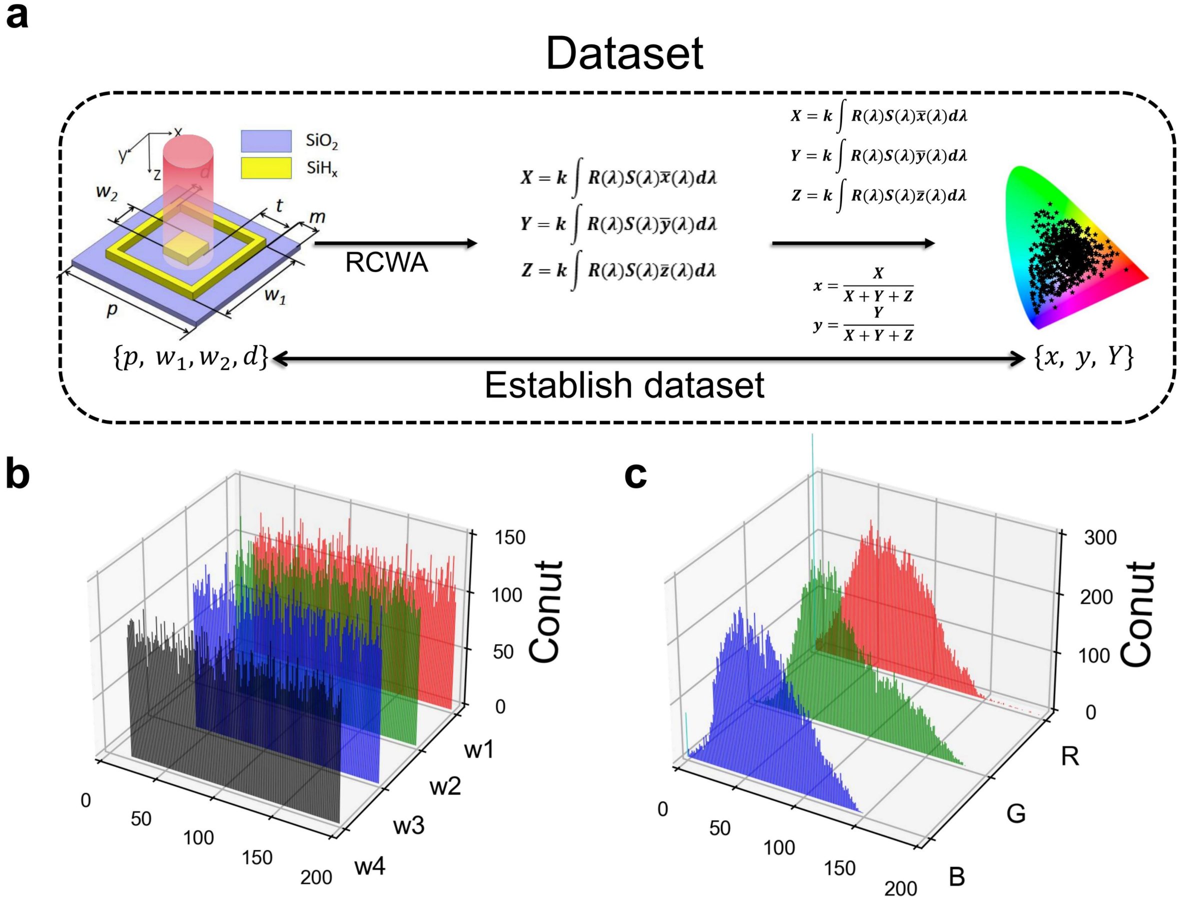

Fig. S2. Information about the dataset. a, Structure parameters are generated by random numbers. The data set is built by calculating the reflected spectrum using RCWA and then computing the chromaticity coordinates. **b**, Statistical histograms of the different structural parameters in the dataset. **c**, Statistical histograms of the colors corresponding to different structures in the dataset.

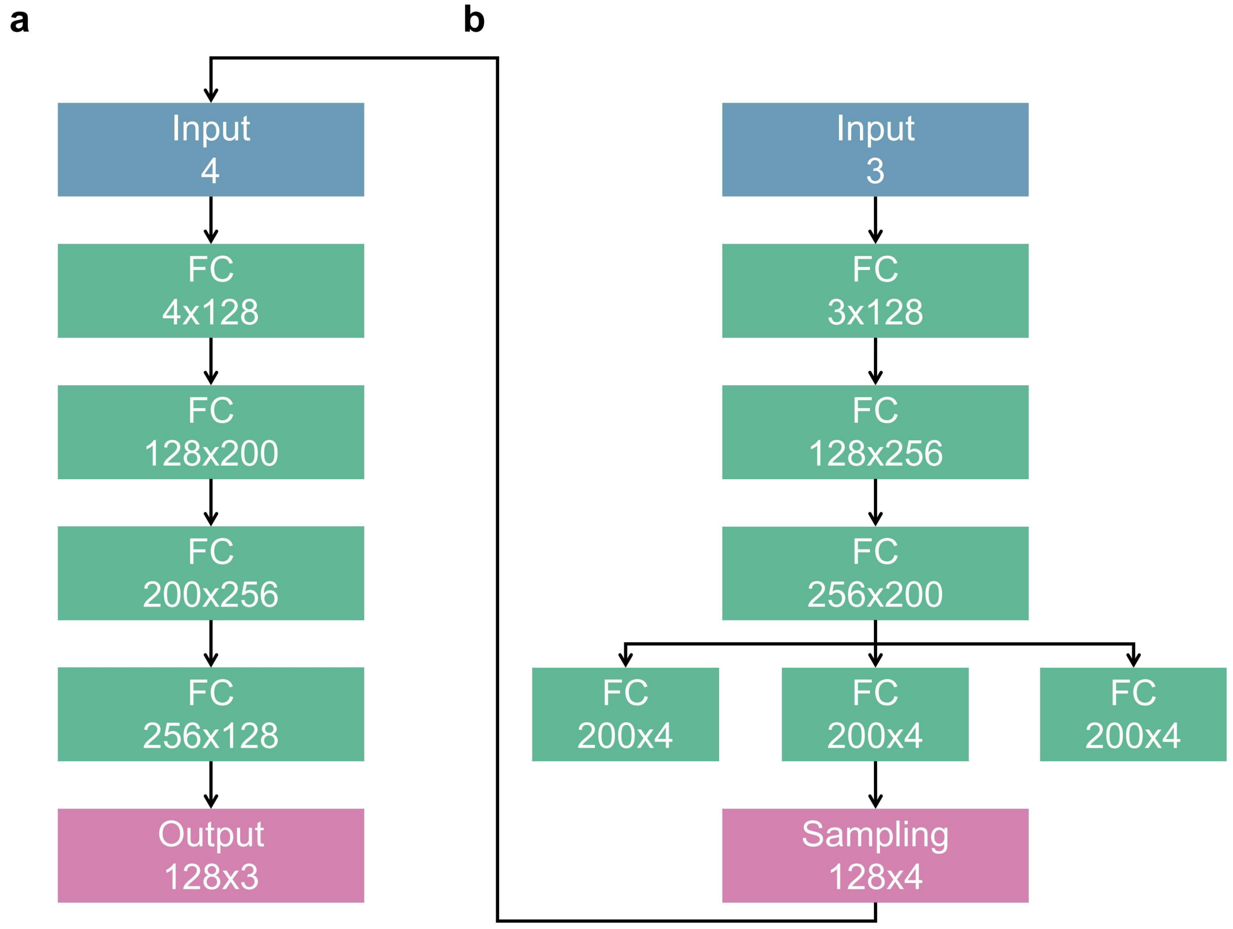

Fig. S3. Network structure diagram. **a**, Schematic of the pre-trained network. It takes four structural parameters $(\boldsymbol{w_1}, \boldsymbol{w_2}, \boldsymbol{w_3}, \boldsymbol{w_4})$ as input and predicts the corresponding chromaticity coordinates $(\boldsymbol{x}, \boldsymbol{y}, \boldsymbol{Y})$. The network comprises four hidden layers with 128, 200, 256, and 200 nodes. **b**, Schematic of the MDN. It receives chromaticity coordinates as input and predicts a mixture Gaussian distribution for the corresponding structural parameters, with each Gaussian component produced by a separate network. The sampled values are subsequently passed through the pre-trained network for further training step.

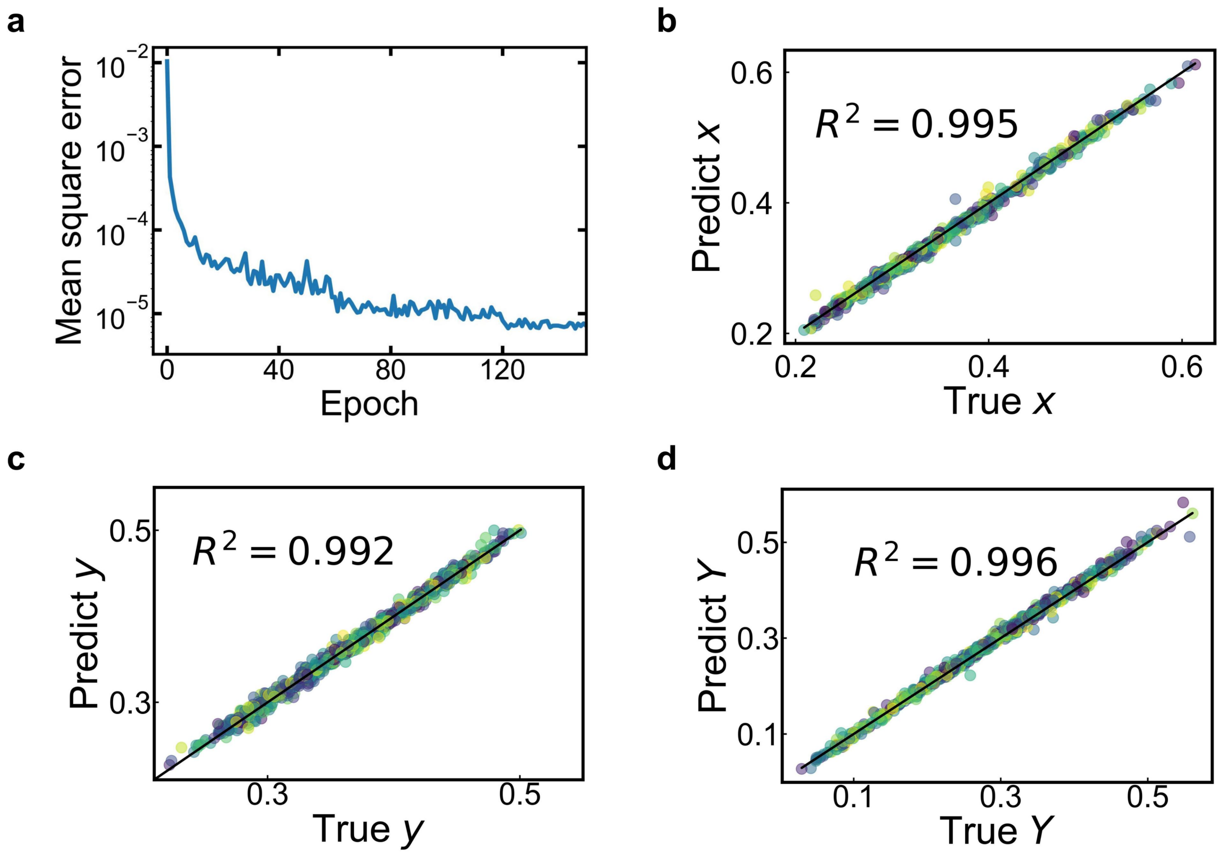

Fig. S4. Training results of the pretrained network. **a**, Training loss curve of the pretrained network. **b-d**, Scatter plots of the true coordinates $(\boldsymbol{x}, \boldsymbol{y}, \boldsymbol{Y})$ and the predicted coordinates $(\boldsymbol{x}, \boldsymbol{y}, \boldsymbol{Y})$ in the CIE 1931 color space.

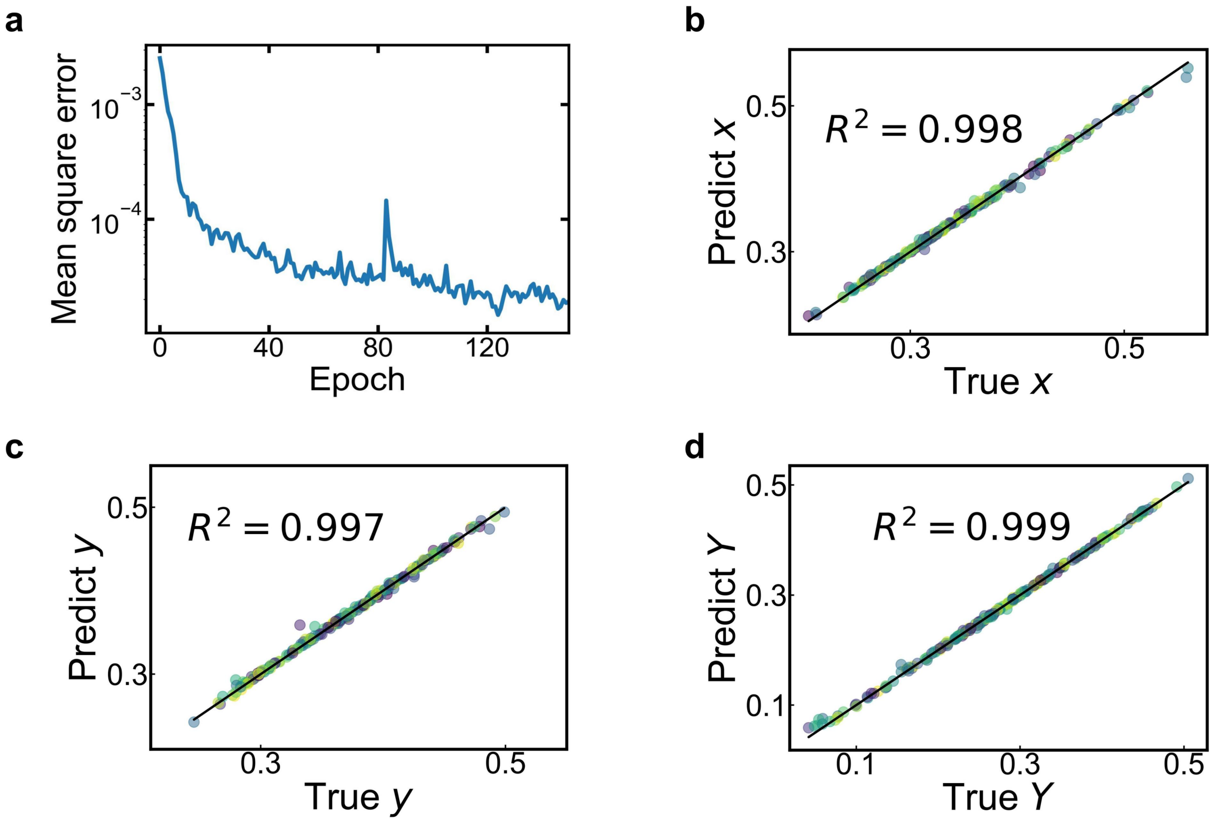

Fig. S5. Training results of the MPSN. a, Training loss curve of the MPSN. **b-d**, Scatter plots of the true coordinates (x, y, Y) and the predicted coordinates (x, y, Y) in the CIE 1931 color space**.**

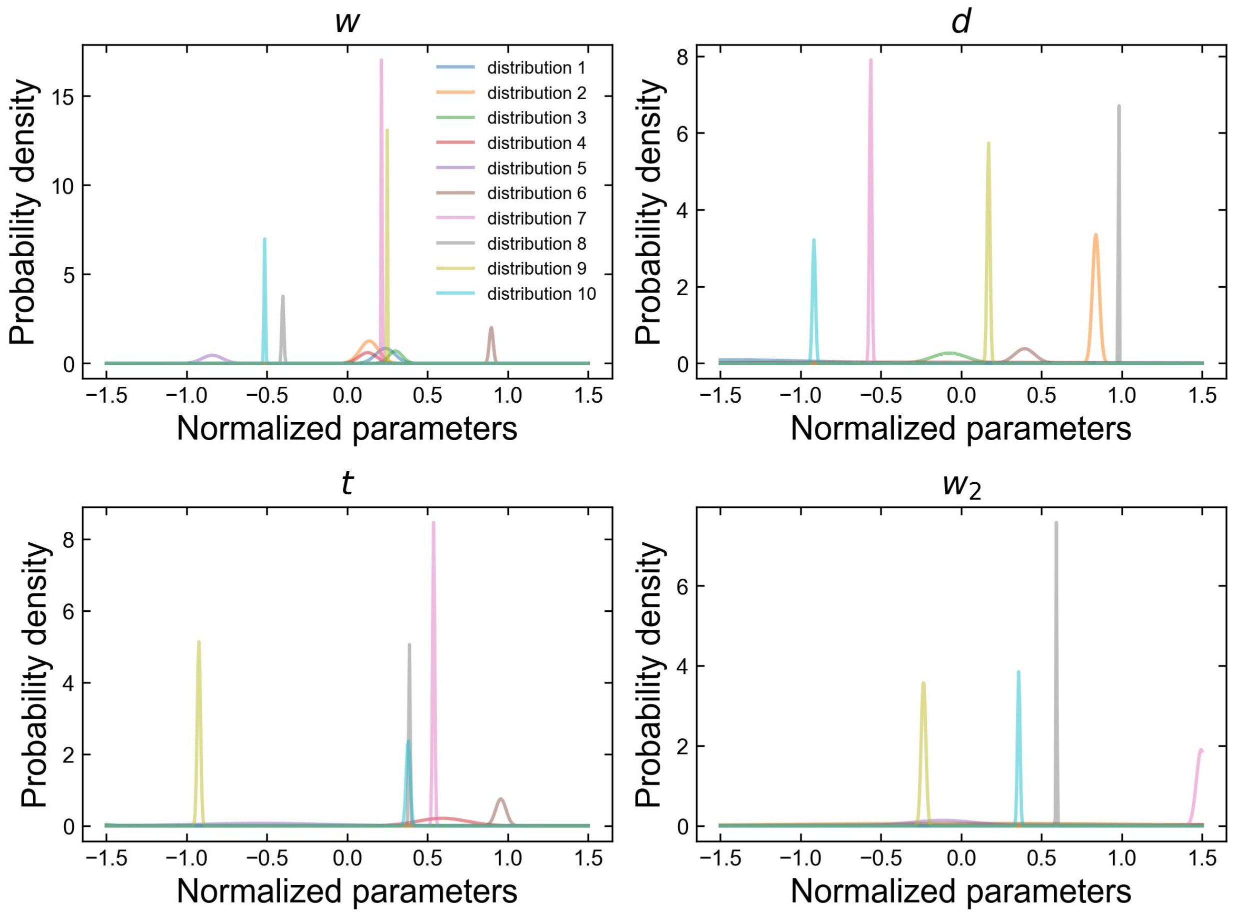

Fig. S6. Output Mixture Gaussion distribution. Predicted mixture of Gaussian distributions for the input color x = 0.31, y = 0.39, Y = 5.23. Curves with distinct colors correspond to different distributions, as indicated in the legend for parameters $\boldsymbol{w}, \boldsymbol{d}, \boldsymbol{t}, \boldsymbol{w_2}$.

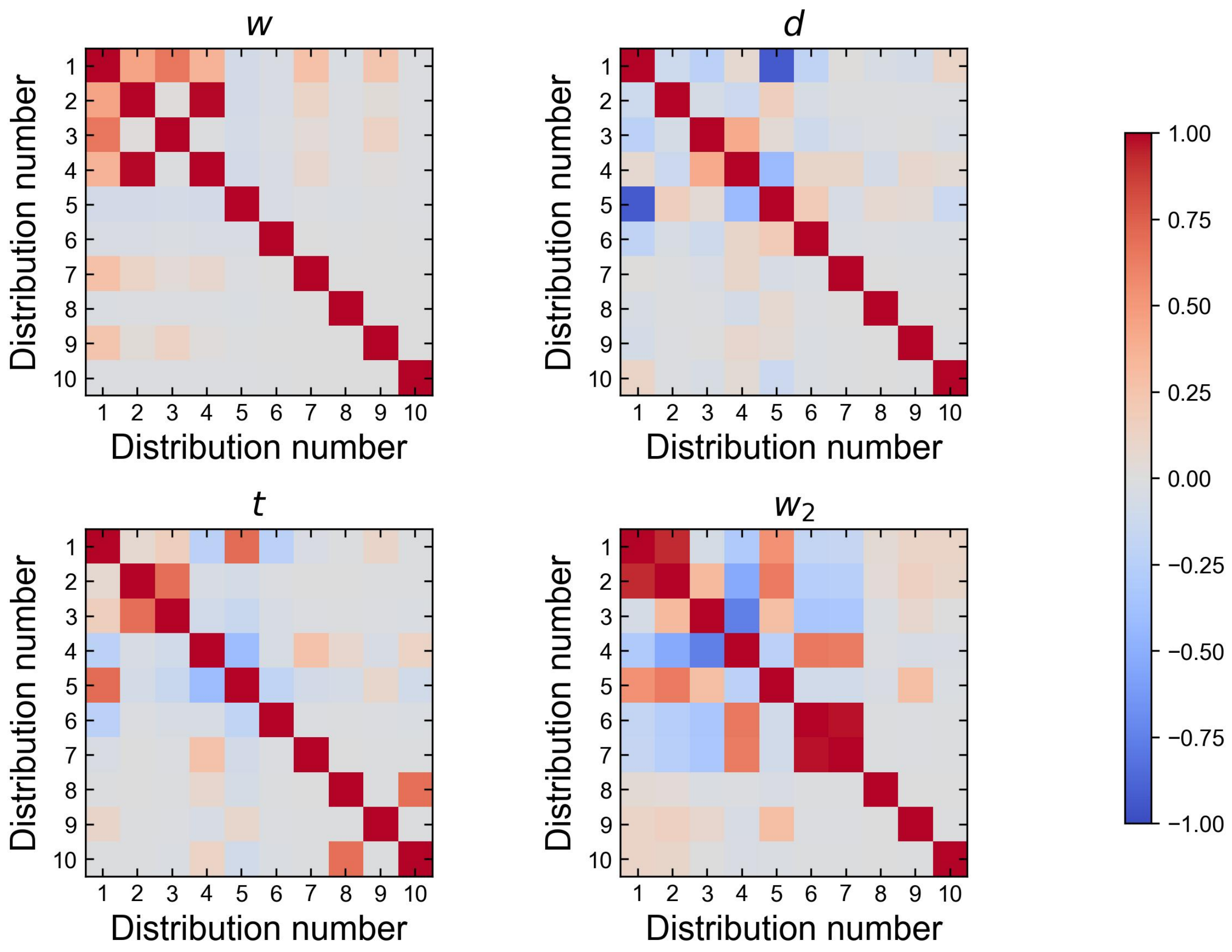

Fig. S7. Pearson correlation coefficient matrix for ten Gaussian components. Warmer colors indicate stronger positive correlations, while cooler colors denote stronger negative correlations. Notably, strong positive correlations are attributed to components with vanishingly small π weights, resulting in near-zero probability density.

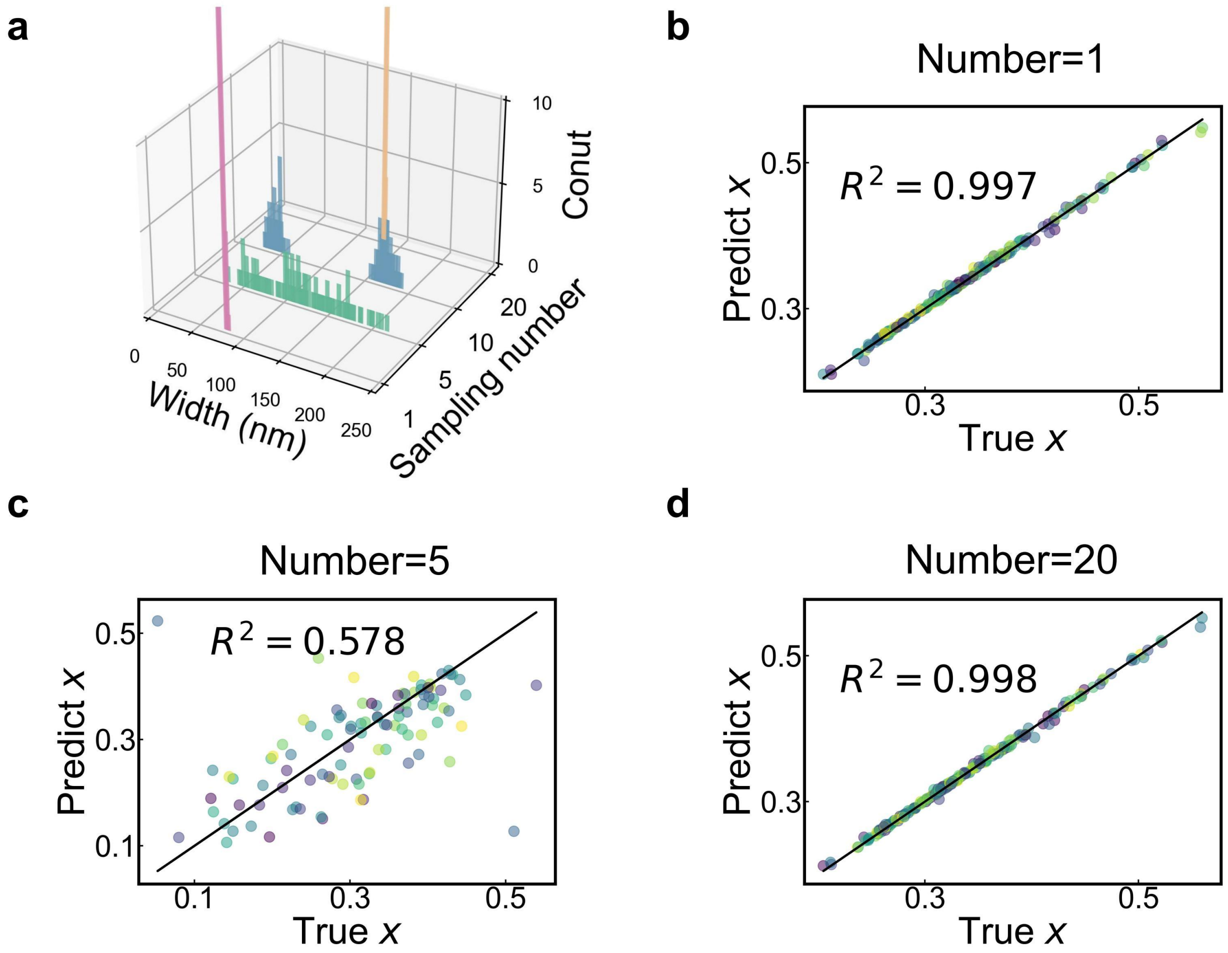

Fig. S8. Comparison of the accuracy and diversity of different sampling numbers. a, Diversity of the network for different sampling numbers. **b-d**, Scatter plots of the true coordinates $(\boldsymbol{x}, \boldsymbol{y}, \boldsymbol{Y})$ and the predicted coordinates $(\boldsymbol{x}, \boldsymbol{y}, \boldsymbol{Y})$ in the CIE 1931 color space for different sampling numbers.

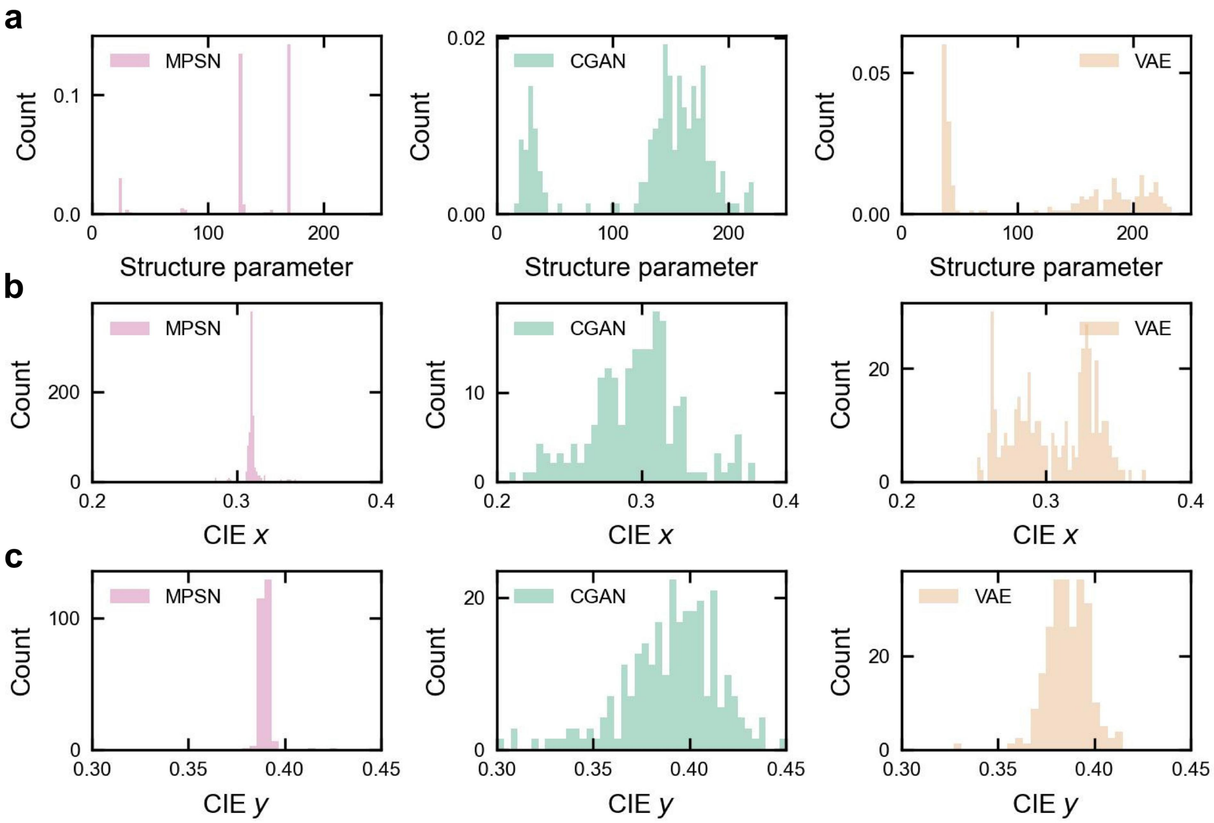

Fig. S9. Statistical analysis of prediction outputs from MPSN, CGAN, and VAE models for input color (0.31, 0.39). a, Statistical histograms of the prediction results for structural parameter *d*. **b-c**, Chromaticity coordinates (x, y) of colors generated by predicted structures.

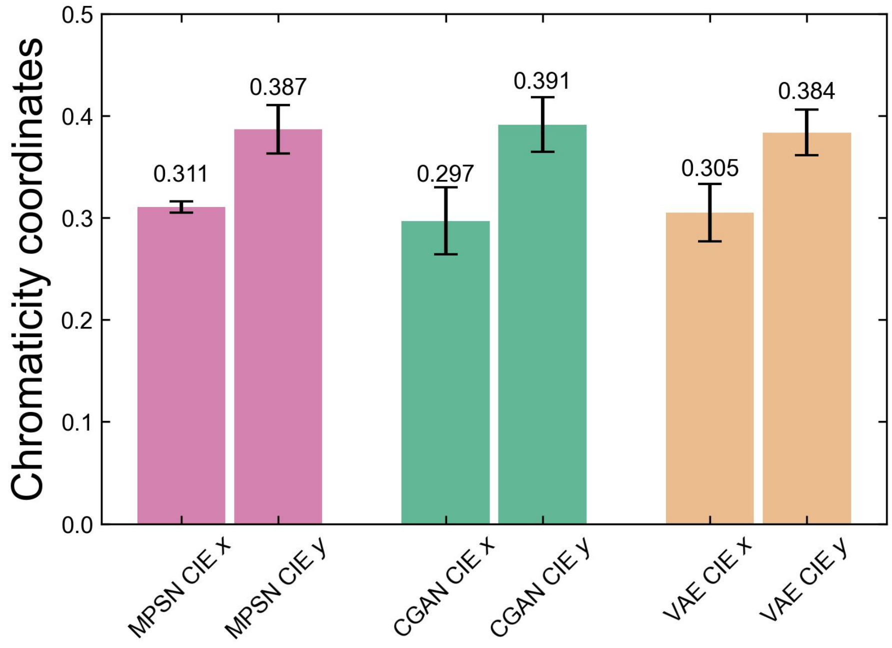

Fig. S10. Bar plot of the colors predicted by MPSN, CGAN, and VAE. Expected values are annotated on bars with 95% confidence intervals shown. Each model is represented by the same color.

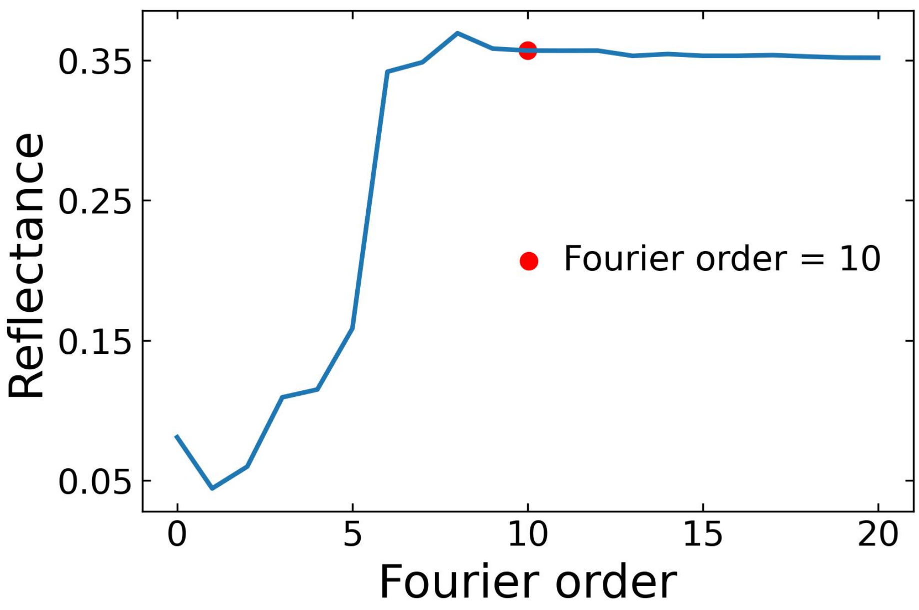

Fig. S11. Convergence Analysis of RCWA. We calculate the convergence curve using the No. 14 structure (green color) from Fig. 4b, with Fourier orders incrementally increased from [0, 0] to [20, 20]. We select [10, 10] as the final computational setting to balance both accuracy and efficiency.

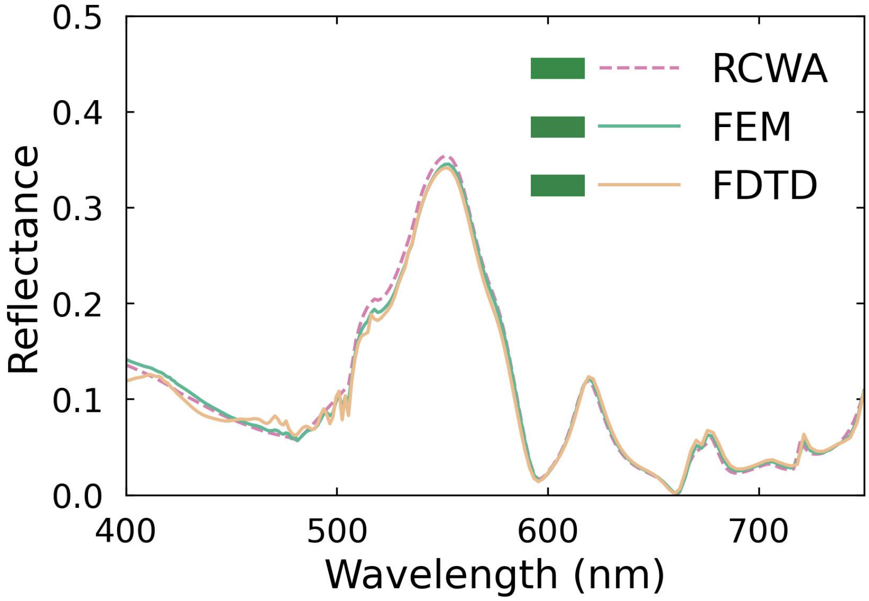

Fig. S12. Accuracy analysis of RCWA. We compute the reflectance of No. 14 structure (green color) from Fig. 4b over the 400–750 nm range for accuracy validation. The pink dashed curve represents the RCWA result with [10, 10] Fourier orders, the green solid curve corresponds to the FEM (COMSOL 6.2) result, and the yellow solid curve denotes the FDTD (Lumerical 2020 R2) result.

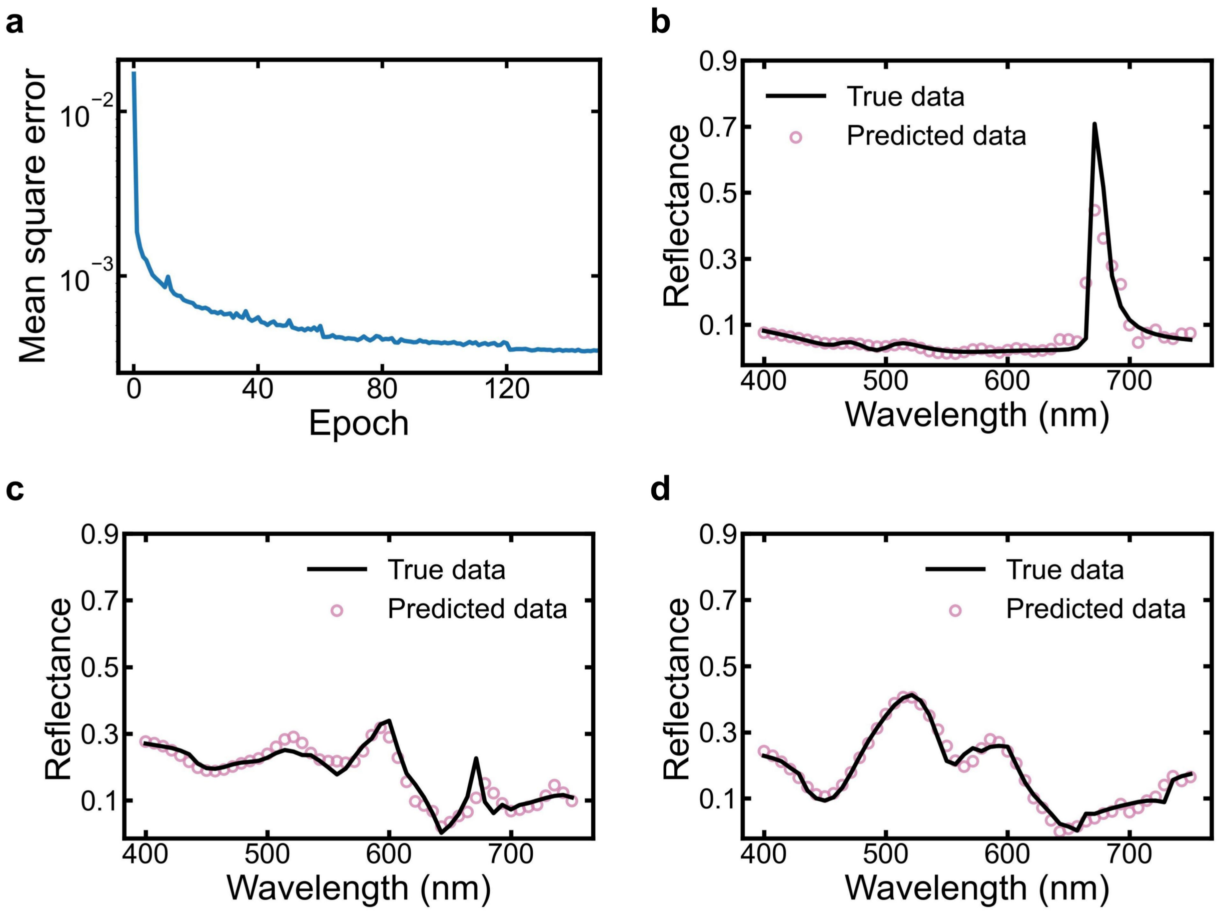

Fig. S13. Training results of the pretrained network. a, Training loss curve of the pretrained network. **b-d**, Spectral prediction results for three selected test cases.

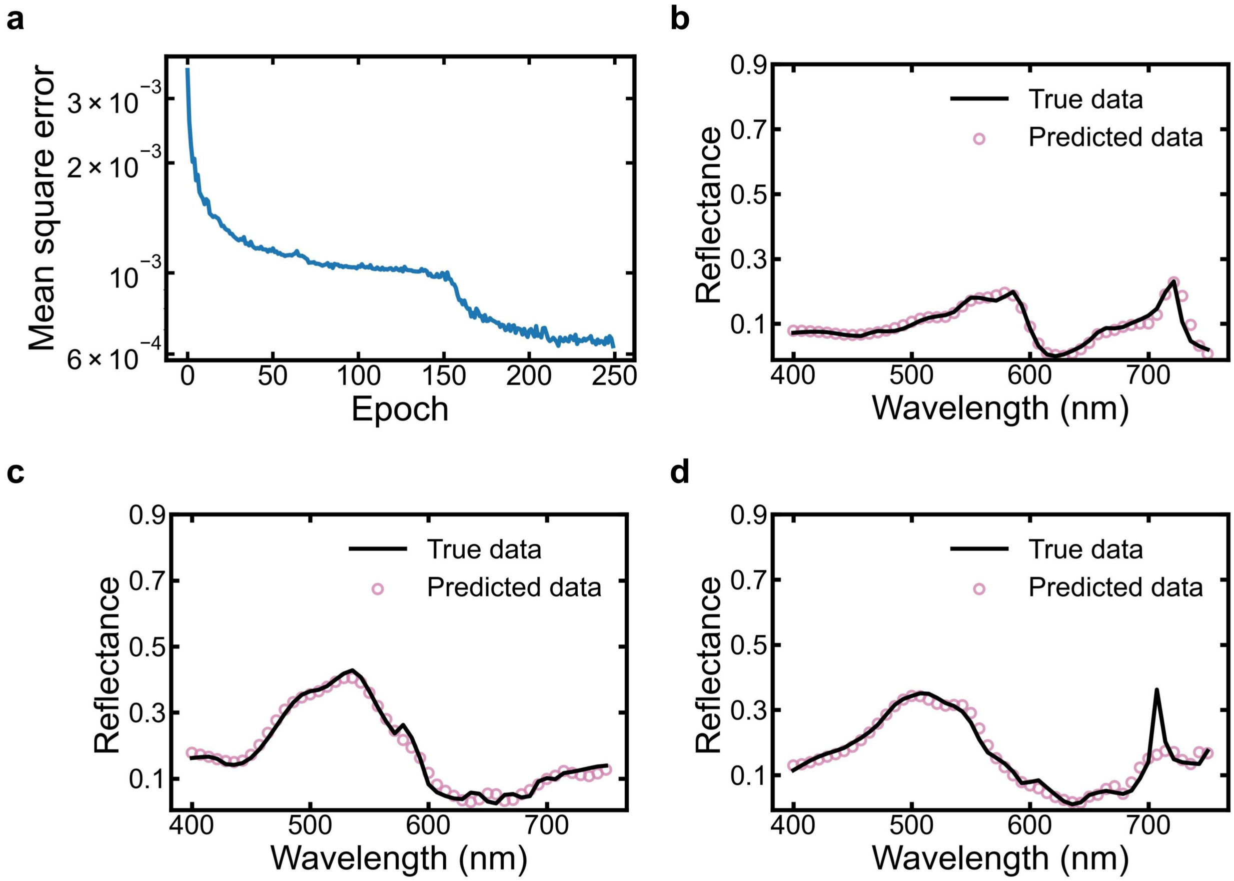

Fig. S14. Training results of the MPSN. a, Training loss curve of the MPSN. **b-d**, Spectral prediction results for three selected test cases.

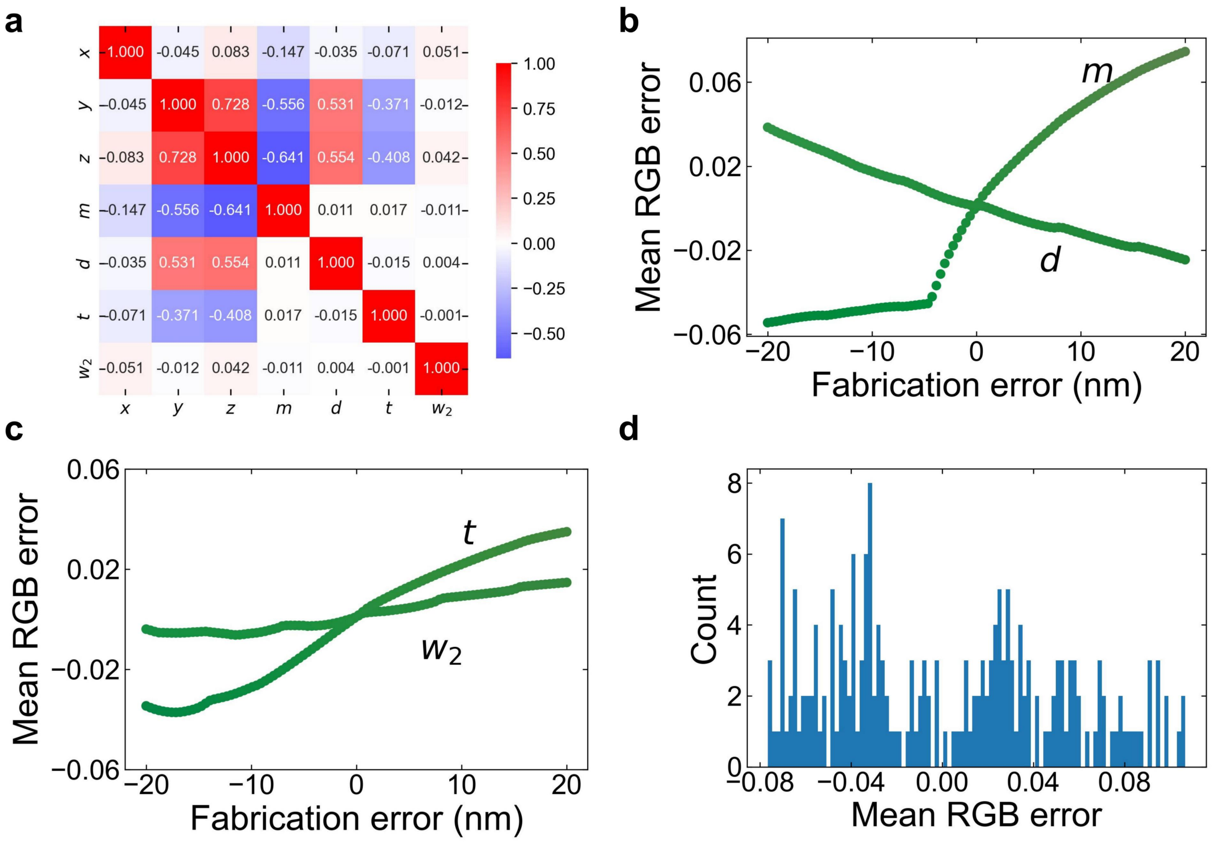

Fig. S15. Fabrication tolerance analysis results. a, Heatmap of the correlation matrix between structural parameters $\boldsymbol{w}, \boldsymbol{d}, \boldsymbol{t}, \boldsymbol{w_2}$ and color coordinates $\boldsymbol{X}, \boldsymbol{Y}, \boldsymbol{Z}$ across the dataset. **b-c**, Mean RGB error profiles under ±20 nm parametric perturbations applied individually to $\boldsymbol{w}, \boldsymbol{d}, \boldsymbol{t}, \boldsymbol{w_2}$, where curve colors represent actual spectral responses. **d**, Statistical histogram of color errors from 200 randomized trials with simultaneous ±20 nm errors on all four parameters.

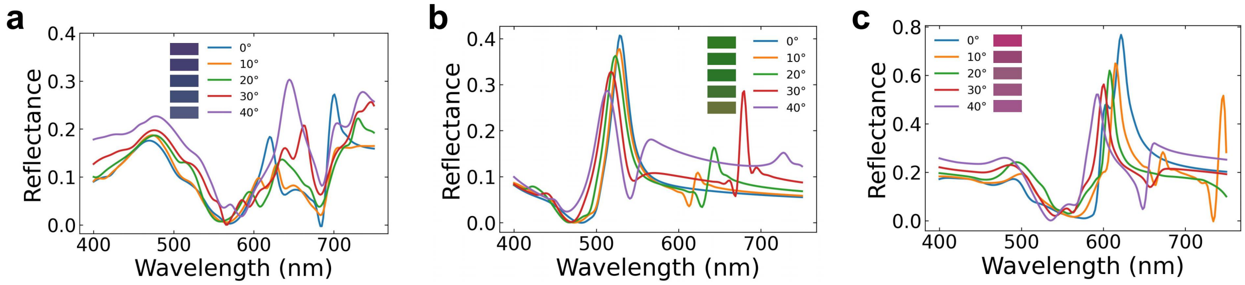

Fig. S16. Angular stability analysis for Color 0, Color 11 and Color 14. Reflectance spectra with corresponding color representations at 0°, 10°, 20°, 30°, and 40° incidence angles. **a**, Color 0, **b**, Color 11, **c**, Color 14.

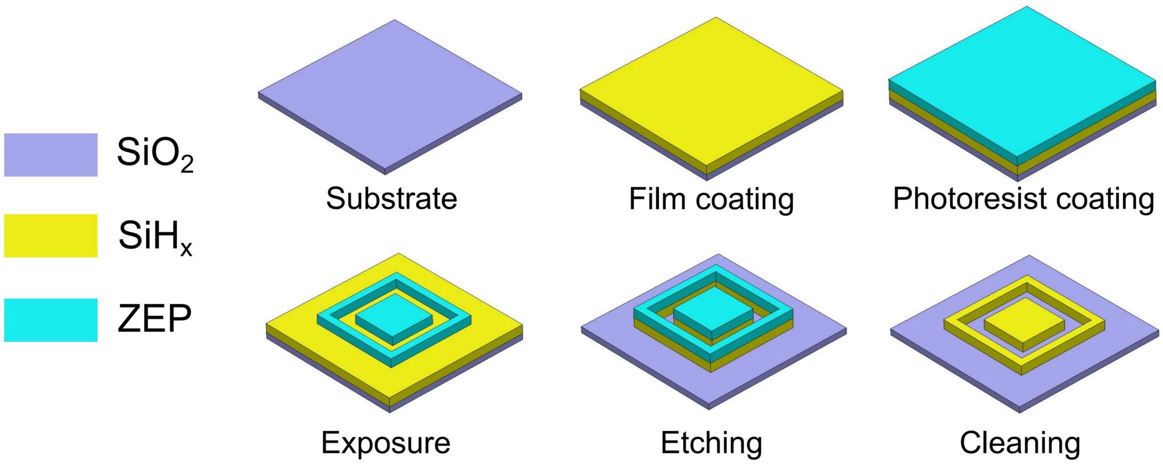

Fig. S17. Fabrication Process. Including the deposition of a SiH_x film on a glass substrate, photoresist spin-coating on the wafer, electron-beam lithography exposure, ICP-RIE etching, and the removal of residual photoresist.

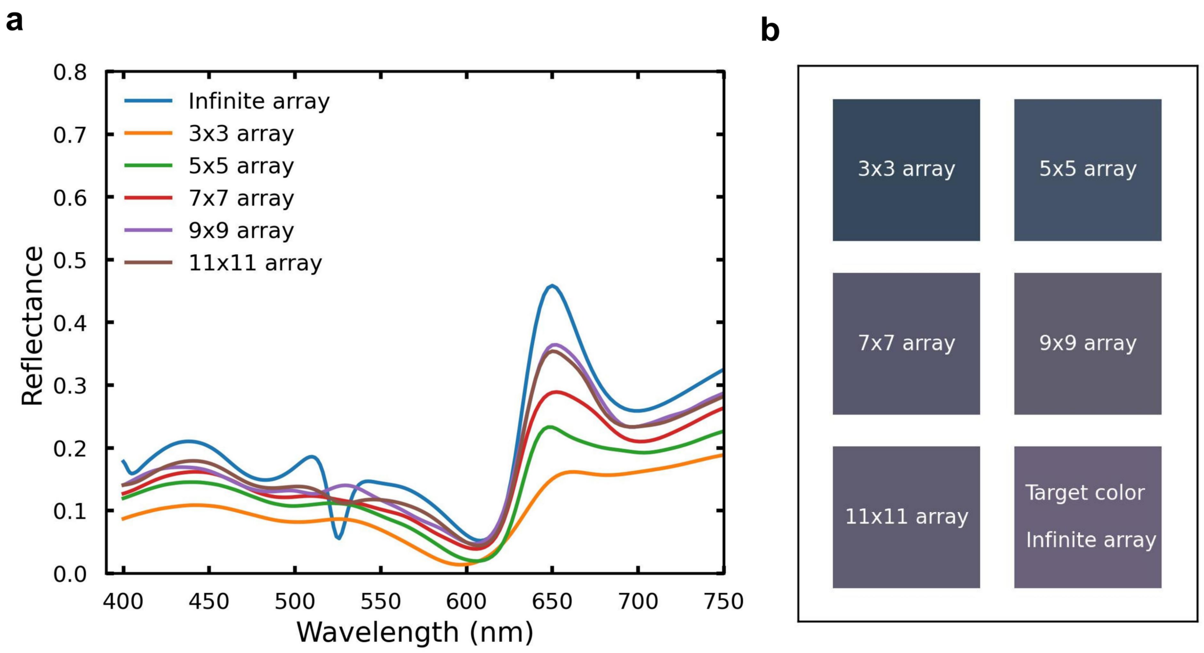

Fig. S18. Color computations for arrays containing different numbers of structure. a, Reflectance in the wavelength range of 400~750 nm, and **b**, reflected colors of arrays containing different numbers of structure.

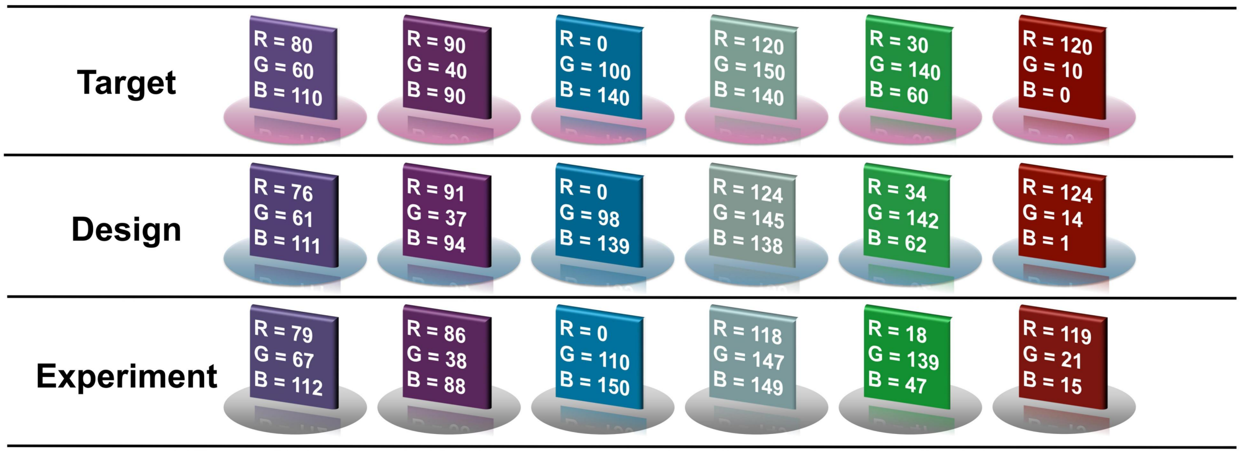

Fig. S19. Comparative results of RGB values for target, designed, and experimental colors. The target color represents the input color to be designed, the designed color corresponds to the output from MPSN, and the experimental color refers to the observed color under optical microscopy.
